# Decoupling the effects of surface morphology and interfacial chemistry on localized electrochemical kinetics in LiCoO_2_ cathodes

**DOI:** 10.1039/d6sc06380b

**Published:** 2026-09-09

**Authors:** Yuxia Guan, Pingshi Wang, Ning Gao, Penghui Liu, Weiwei Cao, Shujiang Ding, Lingjie Meng, Kai Xi, Rui Gao

**Affiliations:** a School of Chemistry, Xi'an Jiaotong University Xi'an 710049 China menglingjie@mail.xjtu.edu.cn kx210.cam@xjtu.edu.cn rui.gao@xjtu.edu.cn; b School of Future Technology, Xi'an Jiaotong University Xi'an 710049 China; c Xi'an Institute of Optics and Precision Mechanics, Chinese Academy of Sciences Xi'an 710119 China; d Engineering Research Center of Energy Storage Materials and Devices, Ministry of Education, National Innovation Platform (Center) for Industry-Education Integration of Energy Storage Technology, Xi'an Jiaotong University Xi'an 710049 China; e Instrumental Analysis Center of Xi'an Jiaotong University Xi'an 710049 China; f Ningxia Advanced Intelligent Sensing and Control Technology Innovation Team, Ningxia Key Laboratory of Bioimaging and Intelligent Diagnostics, North Minzu University Yinchuan 750021 China

## Abstract

Spatial heterogeneity at electrode/electrolyte interfaces critically influences the electrochemical performance and degradation of lithium-ion batteries, yet the respective contributions of surface morphology and interfacial chemistry to localized electrochemical kinetics remain difficult to distinguish experimentally. Existing characterization techniques reveal either material structural transformation or chemical reconstruction but cannot quantitatively decouple their individual effects on local electrochemical behaviour. Herein, we establish a correlative multimodal strategy by integrating scanning electrochemical cell microscopy (SECCM), atomic force microscopy (AFM), Raman spectroscopy, and finite-element modelling to investigate the spatiotemporal evolution of LiCoO_2_ thin films during environmental exposure. Spatially resolved SECCM measurements reveal distinct microdomain-dependent electrochemical heterogeneity despite the initially homogeneous structure of the films. Correlative structural and chemical analyses, together with quantitative simulations, demonstrate that surface morphology and interfacial chemistry govern distinct aspects of the electrochemical response. By independently introducing the roughness factor and the effective lithium-ion diffusion coefficient into the multiphysics model, these two contributions can be quantitatively decoupled, with morphology predominantly determining the current response amplitude and interfacial chemistry governing kinetic limitations. This work provides a distinct strategy for correlating local interfacial evolution with electrochemical function and offers new insights into heterogeneous electrochemical interfaces in advanced energy-storage materials.

## Abstract 2

Correlative SECCM, AFM, Raman spectroscopy, and numerical simulation decouple the effects of surface morphology and interfacial chemistry on localized electrochemical kinetics of LiCoO_2_ cathodes.

## Graphical abstract

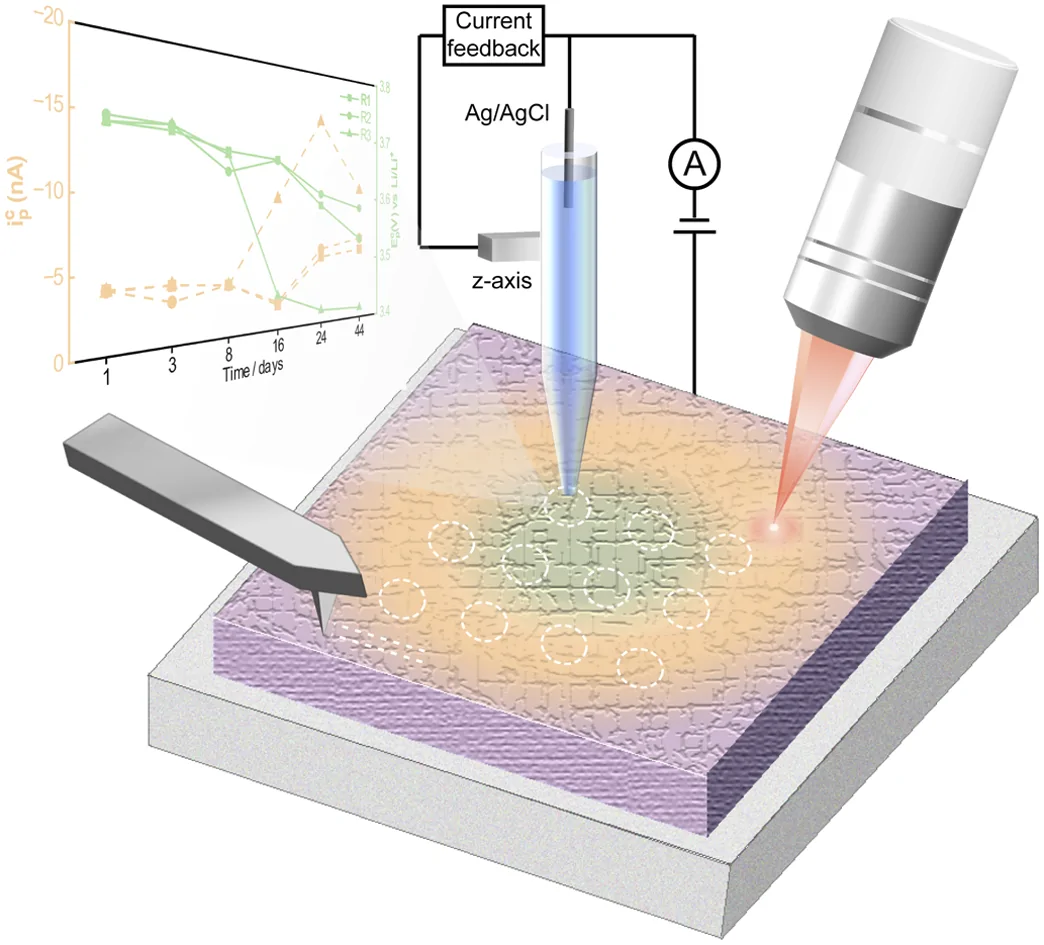

## Introduction

Lithium-ion batteries (LIBs) have become the dominant electrochemical energy-storage technology for portable electronics, electric vehicles, and grid-scale energy systems.^1–3^ Their electrochemical performance relies critically on Li^+^ transport and interfacial charge-transfer processes at the electrolyte/cathode interface, where local reaction kinetics ultimately influence energy efficiency, rate capability, and lifetime.^4–8^ Although cathodes are often treated as compositionally and structurally uniform from the perspective of the full electrode, their electrochemical response is intrinsically heterogeneous across nano- and microscale domains. Spatial heterogeneities in defect density, surface termination, stress state, and chemical composition can induce substantial disparities in Li^+^ insertion behaviours across a single electrode. More importantly, such local variations are not static. Exposure to reactive environments and subsequent surface reconstruction can continuously reshape both the morphology and chemistry of the electrode/electrolyte interface.

Considerable efforts have been devoted to visualizing structural and chemical heterogeneity in battery electrodes using advanced characterization techniques,^9–12^ including X-ray photoelectron spectroscopy (XPS)^13,14^ and transmission electron microscopy (TEM).^15,16^ These approaches have substantially advanced the understanding of surface reconstruction, phase evolution, and chemical degradation. Nevertheless, they predominantly provide structural or compositional information and generally require either ultra-high-vacuum environments, sophisticated sample transfer procedures, or indirect interpretation of electrochemical behaviour.^17,18^ Due to the difficulty in directly correlating structural features with localized kinetic measurements, the respective contributions of these two factors remain difficult to distinguish experimentally. Furthermore, as these morphological and chemical changes occur simultaneously and continuously during kinetic surface reconstruction, it remains difficult to determine whether an apparent change in local electrochemical activity originates primarily from morphological amplification of the active interface or from a genuine deterioration of interfacial transport. Therefore, there is a critical need to quantitatively decouple the individual roles of surface morphology and interfacial chemistry in governing the spatiotemporal evolution of local electrochemical kinetics.

Scanning electrochemical cell microscopy (SECCM) has recently emerged as a powerful tool for probing localized electrochemical activity with high spatial resolution by confining electrochemical measurements within a microscopic electrolyte meniscus at the tip of one pipette.^19–25^ Recently, it has been applied to probe Li^+^ (de)intercalation in individual LiFePO_4_ (ref. 26–30) and LiMn_2_O_4_ (ref. 31 and 32) particles, as well as in Li_4_Ti_5_O_12_ (ref. 33) and LCO^34^ film electrodes, revealing correlations between intrinsic material properties and their electrochemical behaviours.^35^ However, while SECCM maps the spatial distribution of electrochemical reactivity, it is still challenging to unambiguously assign the physical or chemical origins of such heterogeneity with one analytical method. To establish direct structure–activity relationships, electrochemical mapping needs to be correlated with complementary techniques capable of interrogating the exact same microdomain. To explicitly address the challenge of decoupling morphological and chemical factors, atomic force microscopy (AFM) and Raman spectroscopy present an ideal combination. AFM provides quantitative information on local surface topography and structural transformation,^24,36,37^ whereas Raman spectroscopy sensitively probes phase transitions and interfacial chemical states.^38–40^ Integrating these modalities offers a unique opportunity to directly correlate morphology, chemistry, and electrochemical kinetics within identical microdomains.

Here, we employ LiCoO_2_ (LCO) thin films as a model system and integrate SECCM with co-localized AFM and Raman spectroscopy to investigate how subtle initial variations evolve into spatiotemporal electrochemical heterogeneity during ambient storage by tracking the coupled evolution of local electrochemical response, surface morphology, and near-surface phase/chemical composition across subtle microdomains. Combining experimental characterization with finite element numerical simulations, we quantitatively analysed the independent contributions of morphology-induced changes in effective interfacial area and chemically induced changes in Li^+^ transport. Specifically, we demonstrate that morphological changes predominantly regulate the electrochemically active interface and govern current magnitude, whereas interfacial chemical reconstruction progressively limits lithium-ion transport and dominates the kinetic degradation reflected by peak-potential shifts. Beyond elucidating the degradation mechanism of LCO, this work establishes a robust strategy for quantitatively resolving the spatiotemporal coupling among local structure, chemistry, and electrochemical function, providing new insights into heterogeneous electrochemical interfaces and guiding the rational design of next-generation energy-storage materials.

## Results and discussion

### Spatiotemporal divergence of local lithium-ion kinetics revealed by SECCM

Thin-film LCO provides an ideal model system because of its macroscopically uniform morphology, enabling intrinsic local heterogeneity generated during surface reconstruction to be investigated without the complexity associated with composite porous electrodes.^41,42^ In this work, the LCO films were fabricated *via* radio frequency (RF) magnetron sputtering in a custom-built vacuum chamber,^42,43^ yielding a dense layer composed of particles with uniform size and a thickness of ∼400 nm, which exhibits an apparently homogeneous surface after deposition (Fig. S1). However, such macroscopic uniformity does not necessarily represent the intrinsic electrochemical behaviour of individual surface domains. To visualize the evolution of local lithium-ion kinetics during environmental evolution, SECCM was employed to perform spatially resolved cyclic voltammetry measurements on representative microdomains of the LCO film (Fig. 1). Three characteristic regions (R1–R3) were selected based on their distinct optical contrast after air exposure, representing different stages of surface reconstruction (Fig. S2). Time-dependent SECCM measurements were subsequently performed at selected exposure intervals (day 0, 3, 8, 16, 24, and 44) using a ∼4 µm-diameter nanopipette, which can form a confined electrochemical cell after approaching the LCO surface (Fig. S3). The SECCM instrumentation and measurement configuration are shown in Fig. S4.

**Fig. 1:**
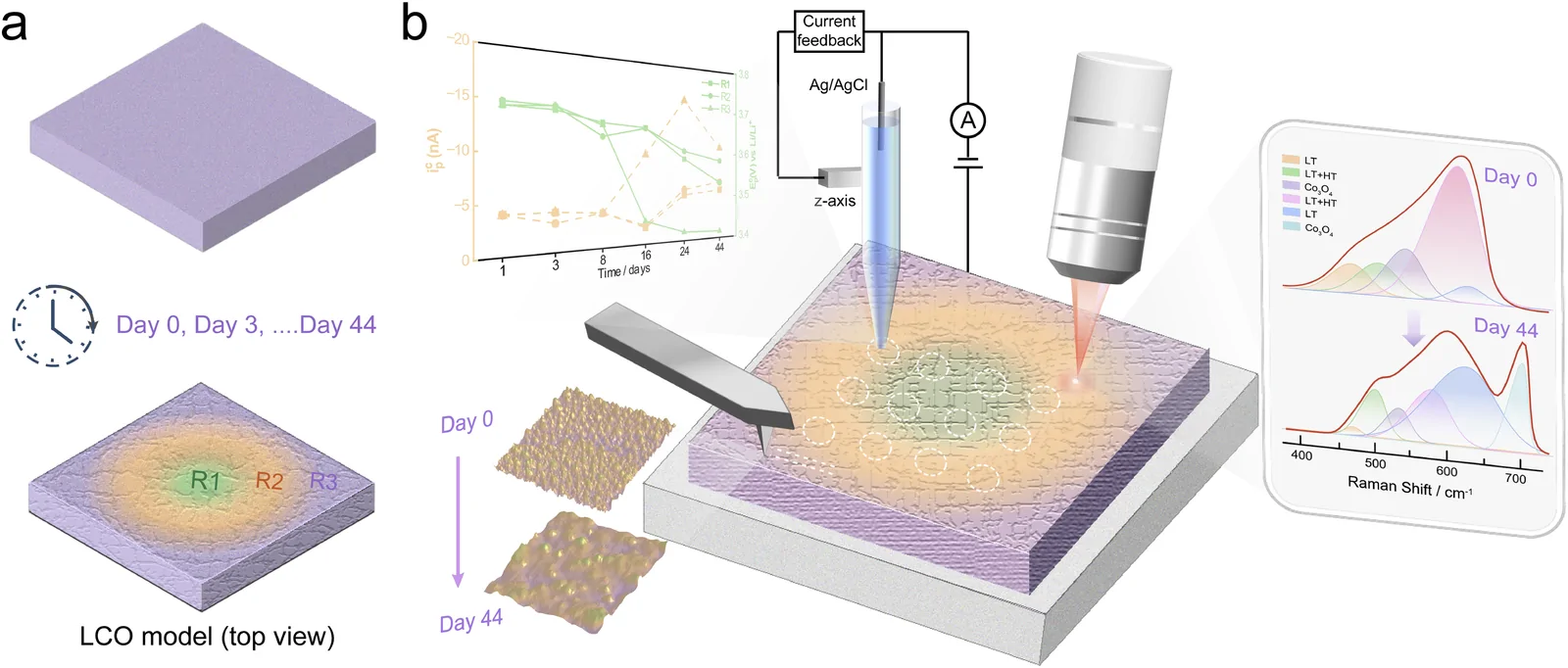
Schematic illustration of the correlative multi-modal characterization strategy employed to interrogate the spatiotemporal heterogeneity of LCO films. (a) Structural transformation of distinct LCO domains. (b) Schematic of hopping-mode SECCM, utilizing a nanopipette filled with 1 M LiCl to perform spatially resolved electrochemical measurements on R1, R2, and R3 domains, coupled with co-localized Raman spectroscopy and AFM for correlative structural and chemical analysis.

For the freshly deposited LCO film, all three domains exhibit comparable cyclic voltammetric responses, characterized by a pair of broad anodic and cathodic peaks located at 3.89 and 3.69 V *vs.* Li/Li^+^, corresponding to the reversible lithium deintercalation/intercalation process (Fig. S5).^44^ This comparable electrochemical activity confirms the intrinsic uniformity of the initial film. Nevertheless, an obvious spatial heterogeneity gradually emerges upon environmental exposure. With increasing exposure time, the anodic peak gradually diminishes and eventually becomes indistinguishable, while the cathodic peak exhibits a continuous negative shift. This result indicates that the local Li^+^ kinetics deteriorate heterogeneously across different microdomains during the LCO degradation.

To further examine the electrochemical reversibility of individual microdomains, repeated CV measurements were performed using SECCM (Fig. S6). A distinct electrochemical activation process was observed. Particularly after 16 days of exposure, the anodic peak, which is barely discernible in the second cycle, reappeared after continuous cycling (*e.g.*, by the 15th cycle). This indicates that the lithium (de)intercalation capability decreases due to surface lithium loss during prolonged exposure, requiring multiple electrochemical cycles to be reactivated. Notably, the response current showed a continuous increase with the cycle number. We therefore define a retention efficiency to quantitatively evaluate the cycling stability, which is defined as the ratio of the cathodic peak current of the *n*-th cycle (*n* > 2) to that of the second cycle (*n* = 2). As a result, the normalized local retention efficiencies (Fig. 2a–c, the normalization method is detailed in Fig. S6) consistently exceeded 100% across all three domains at different times. In contrast, this phenomenon is distinct from the typical decay characteristics of conventional battery materials caused by cycling. We speculate that this behaviour may be associated with the relatively high wettability of the LCO surface, which facilitates spreading of the liquid meniscus at the pipette tip, thereby expanding the effective reaction area. This interpretation is supported by the contact angle measurements shown in Fig. S7. On day 0, the R3 domain exhibited high wettability, with the average contact angle decreasing rapidly from 46.6° to 25.6° within 5 s. By day 44, however, the contact angle decreased only from 84.05° to 73.4°. This difference in wettability, resulting from surface structural and compositional changes induced by continuous exposure, is consistent with the slowing growth rate of the current observed in the R3 domain (Fig. 2c). Despite the current gain from droplet spreading, the retention efficiency of each domain eventually shows a trend of increasing and then decreasing over long-term exposure, likely due to surface cracking, structural collapse, and byproduct accumulation. In particular, the R3 domain exhibited evident performance decay as early as day 8. This specific early decay will be discussed in detail later in conjunction with relevant data.

**Fig. 2:**
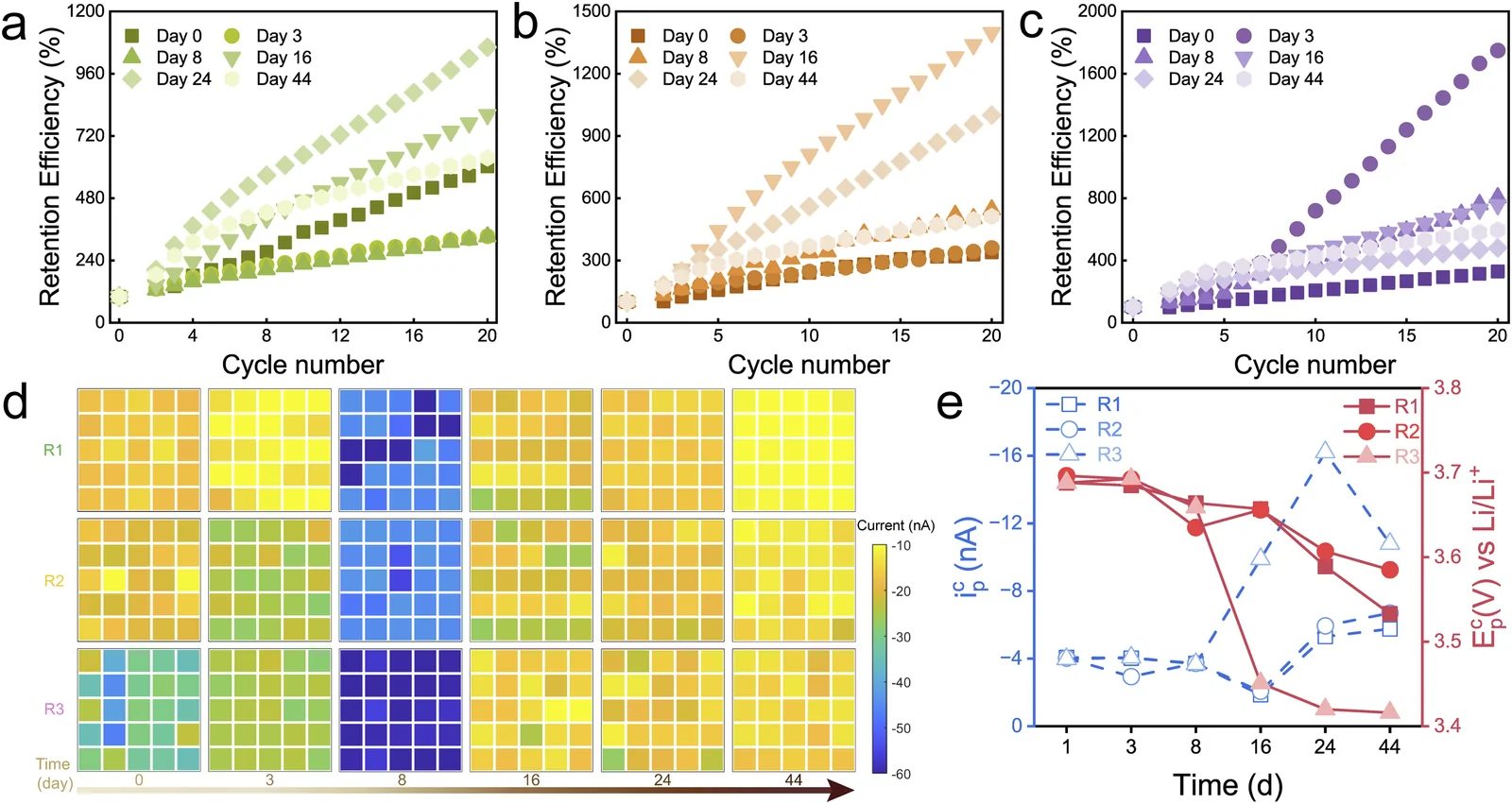
Electrochemical heterogeneity of LCO film microdomains. (a–c) Capacity retention of the (a) R1, (b) R2, and (c) R3 microdomains of the LCO film after 20 voltammetric cycles with extended air exposure (day 0, 3, 8, 16, 24, and 44). (d) SECCM maps of the intercalation current at 3.69 V *vs.* Li/Li^+^ acquired over different microdomains at different times. Each image consists of a 5 × 5 pixel array covering a scan area of 60 × 60 µm^2^. (e) Line plots of the cathodic peak potential and peak current corresponding to the R1, R2, and R3 microdomains.

Fig. 2d displays the spatiotemporal evolution features of electrochemical activity in each domain (mapped based on the intercalation current at 3.69 V *vs.* Li/Li^+^). The colour uniformity within each domain confirms the homogeneous distribution of local electrochemical properties, and the signal intensity in all three domains follows a trend of initially increasing and subsequently decreasing. The underlying mechanism for this trend can be reasonably explained by the changes in kinetic parameters shown in Fig. 2e. Although the peak current, *i*^c^_p_ maintained an increasing trend for a considerable exposure period (*e.g.*, up to day 24 for the R3 domain) due to the aforementioned droplet spreading effect, the cathodic peak potential, *E*^c^_p_ gradually shifted negatively over time. This significant negative shift signifies sluggish kinetics for the Li^+^ intercalation reaction and intensified interfacial polarization, causing the current recorded at the potential of 3.69 V to decrease substantially as it deviates from the peak potential window. This competition between physical wetting spreading (leading to increased *i*^c^_p_) and chemical surface passivation (leading to the negative shift of *E*^c^_p_) clearly reveals the complexity of the surface reconstruction.

These observations raise an important mechanistic question regarding how the respective contributions of surface morphology evolution and interfacial chemical reconstruction can be quantitatively separated. To address this challenge, subsequent correlative AFM and Raman analyses are performed to independently track morphological and chemical reconstruction within the same microdomains.

### Surface morphology evolution regulates the apparent electrochemical interface

The SECCM measurements reveal an unexpected enhancement of local current during the early stage, suggesting that electrochemical accessibility may evolve differently from intrinsic reaction kinetics. To elucidate the morphological origin of this behaviour, AFM was employed to characterize the evolution of surface morphology in the same electrochemical microdomains. Although the as-deposited film appears relatively uniform at the macroscopic scale (Fig. 3a), Fig. 3b shows that the morphology mapping of different microdomains from day 0 to 44 reveals relatively smooth surfaces in freshly prepared LCO films, with slight nanoscale height fluctuations remaining. With time going by, all three domains gradually develop increasingly rough surface features accompanied by particle coarsening and crack formation. These morphological changes are not spatially uniform. The R3 domain exhibits the most obvious crack propagation and surface reconstruction, whereas R1 undergoes comparatively limited morphological changes, with R2 displaying an intermediate degree of reconstruction.

**Fig. 3:**
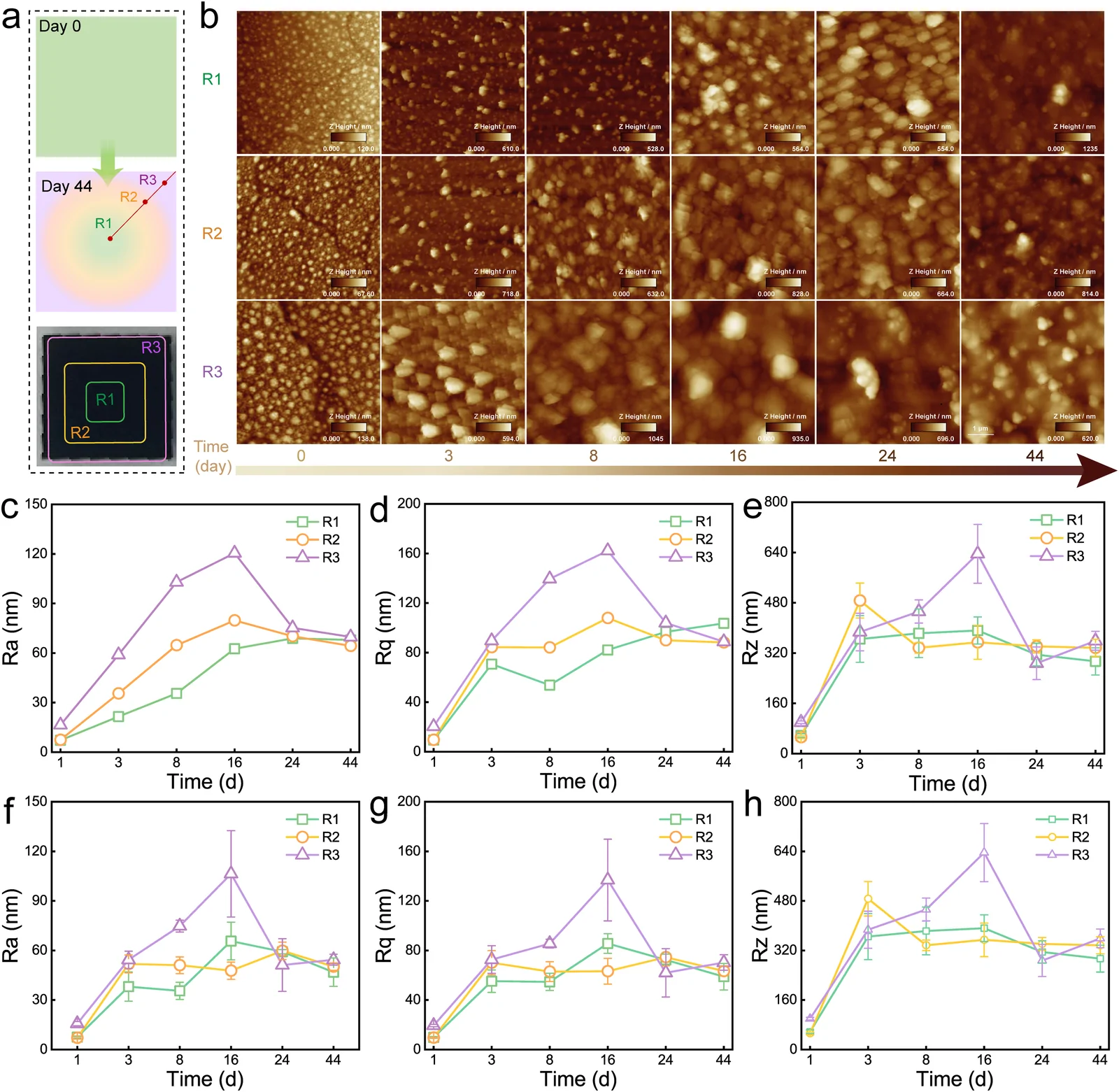
Spatiotemporal evolution of the surface morphology of LCO film domains. (a) Schematic illustration of three characteristic micrometer-scale domains (R1, R2, and R3) with distinct morphological features formed in the LCO film over time. (b) Representative 2D AFM surface topography images of the R1, R2, and R3 micrometer-scale domains over time (day 0–day 44). (c–e) Evolution of global surface roughness parameters extracted from AFM images as a function of air-exposure time, including (c) the arithmetic mean roughness (*R*_a_), (d) root-mean-square roughness (*R*_q_), and (e) ten-point height (*R*_z_). (f–h) Evolution of local surface roughness parameters as a function of air-exposure time, obtained from line profile analysis, including (f) *R*_a_, (g) *R*_q_, and (h) *R*_z_. Line profiles were obtained from three 5 µm-long line segments selected from each AFM image, and the average values with standard deviations were used to characterize the local roughness distributions of the micrometer-scale domains.

Across the three domains, the surface particles progressively coarsen and agglomerate, with the extent of surface reconstruction and particle coarsening following the order R3 > R2 > R1. Although there are clear differences in evolution rates, if the exposure time is further extended, the surface morphology of the R1 and R2 domains will eventually approach the stable state presented by R3. Combined with the 3D AFM images in Fig. S8, it can be further seen that these morphological changes directly regulate the local electrochemical response. With increasing exposure time, on the one hand, the coarsening and agglomeration of surface particles significantly increase the surface roughness, making the real surface area between the meniscus electrochemical cell and the electrode significantly larger than its morphology projection area; on the other hand, the increase in surface cracks and the expansion of their width allow the electrolyte to penetrate deep along the cracks, thereby enabling more Li^+^ located in the surface and sub-surface layers to participate in the interfacial reaction. The synergistic effect of these two morphology effects provides a reasonable structural explanation for the current signal enhancement observed in the early stage of exposure-induced degradation in the SECCM tests mentioned earlier.

Quantitative roughness analysis further confirms the progressive morphology evolution of the LCO surface. From Fig. 3c–h, the arithmetic mean roughness (*R*_a_), root-mean-square roughness (*R*_q_), and ten-point mean height (*R*_z_) were extracted for quantitative analysis, representing the average height deviation, sensitivity to local peaks and valleys, and extreme vertical surface fluctuations, respectively. Considering the potential statistical errors caused by the severe morphological changes, the global average values (Fig. 3c–e) were analysed in combination with the line profile averages (Fig. 3f–h) and sub-region averages (Fig. S9). This combined analysis reveals that the three microdomains share the same roughness evolution trend. Specifically, the *R*_a_, *R*_q_, and *R*_z_ values of the R3 microdomain increased rapidly within 16 days and then showed an evident decline at day 24, which is consistent with the morphological changes observed in Fig. 3b. The decline starting from day 24 is primarily attributed to the fusion of small-sized particle agglomerates to form larger and relatively flatter agglomerated structures, slightly reducing the overall surface fluctuation. The overall evolution trends of the R1 and R2 microdomains are much milder, which further demonstrates the spatial heterogeneity of the surface morphology evolution across different microdomains. Due to its higher initial defect density, the R3 domain is more susceptible to environmental factors such as H_2_O and CO_2_, leading to the accelerated collapse and roughening of its surface structure, and inducing the rapid degradation of the interfacial electrochemical performance in this domain, consistent with the non-monotonic current evolution discussed above.

Taken together, these results demonstrate that surface morphological changes primarily regulate the electrochemical response through modulating the effective reaction area. This morphology effect operates in parallel with the chemical processes that govern intrinsic reaction kinetics and thus represents a key parameter for quantitatively interpreting the spatiotemporal evolution of electrochemical activity.

### Interfacial chemical reconstruction governs intrinsic lithium-ion transport kinetics

Although AFM analysis demonstrates that surface morphological changes enlarge the electrochemically accessible interface, morphological effects alone cannot account for the continuous negative shift of the cathodic peak potential observed by SECCM. This discrepancy indicates that intrinsic kinetic deterioration may originate from other interfacial evolutions instead of morphological reconstruction. Given this, Raman spectroscopy was systematically performed on all three domains over 1–44 days to explore the surface chemistry transformation (Fig. 4a–c). Overall, the dominant Raman features of LCO are located in the low-wavenumber domain of 400–700 cm^−1^, which is primarily associated with Co–O lattice vibrational modes. The spectra from different microdomains exhibit distinct degrees of peak broadening and feature differentiation, indicating that the near-surface structure is not highly long-range ordered but instead resembles a state with limited crystallinity and substantial defects and local disorder. At the early stage of surface reconstruction, a Raman peak at ∼1088 cm^−1^ was observed in all three microdomains, which became more evident after day 3 and was assigned to the symmetric stretching vibration of CO_3_^2−^.^45^ This feature arises from the formation of Li_2_CO_3_ as a byproduct upon prolonged contact of LCO with H_2_O and CO_2_ in air.^46^ Its relative intensity becomes approximately stable after day 16, indicating the establishment of a Li_2_CO_3_-rich surface layer. By covering surface fissures and forming a poorly Li^+^-conducting passivation layer, the accumulated Li_2_CO_3_ suppresses interfacial Li^+^ transport, consistent with the negative peak shift and kinetic attenuation in Fig. 2e.

**Fig. 4:**
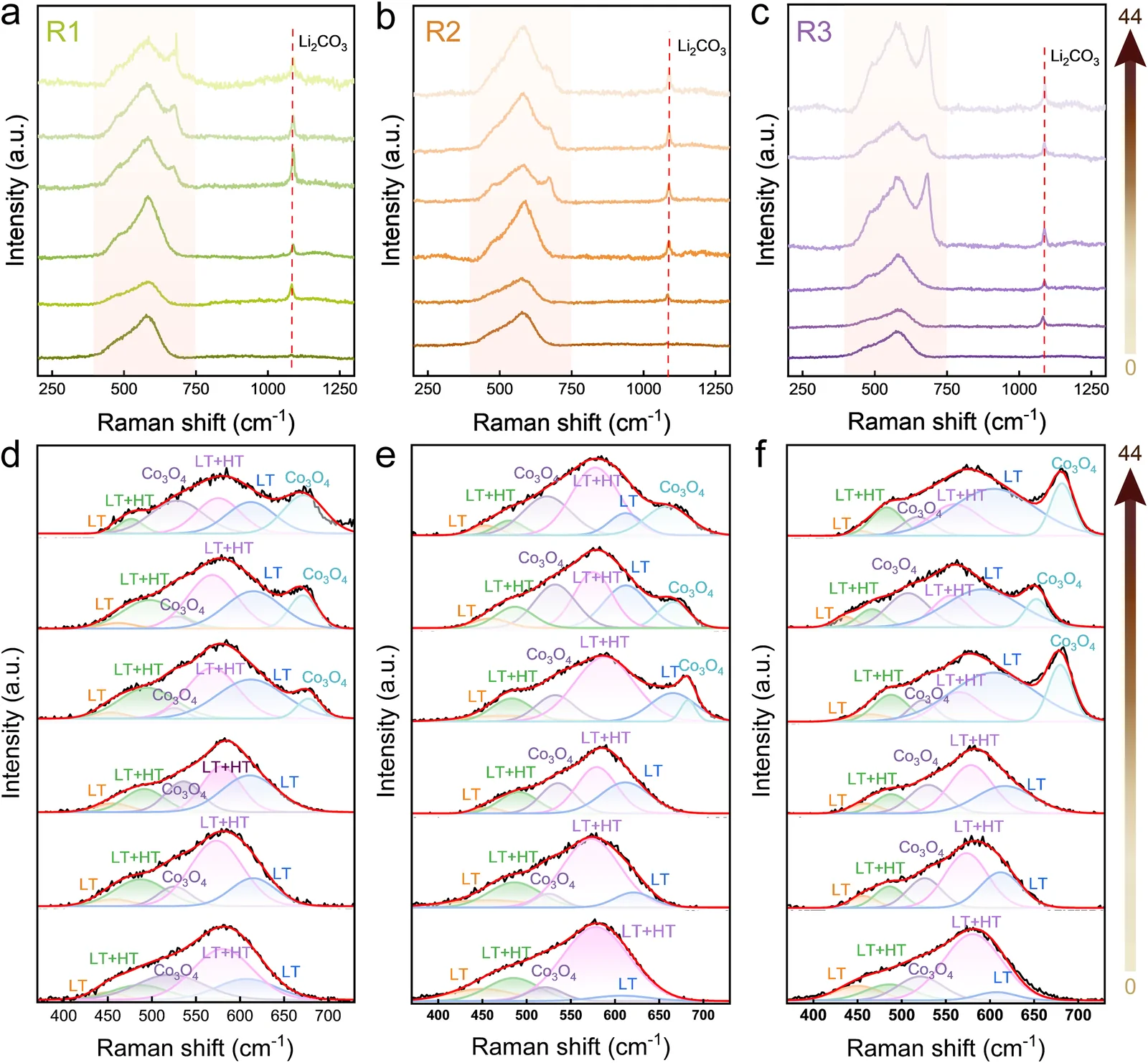
Spatiotemporal evolution of chemical composition and phase structure in LCO film domains. (a) Raman spectra of the R1 domain, (b) R2 domain, and (c) R3 domain at different times. Deconvolution fitting of the Raman spectra in the 400–700 cm^−1^ domain for the (d) R1 domain, (e) R2 domain, and (f) R3 domain, where LT represents the cubic spinel phase and HT represents the hexagonal phase. In each panel, the spectra from bottom to top correspond to the exposure times of day 0, 3, 8, 16, 24, and 44, respectively.

Multi-peak fitting of the spectra shows the coexistence and evolution of different phase structures (Fig. 4d–f). The coexistence and evolution of different phase structures are reflected in the 400–700 cm^−1^ range of the spectrum. The hexagonal (HT) LCO phase exhibits E_g_ and A_1g_ bands at ∼487 and 596 cm^−1^, whereas the cubic spinel-like (LT) LiCoO_2_ shows peaks at ∼450, 485, 591, and 607 cm^−1^.^47^ The broad coexistence of HT and LT features in the as-deposited film confirms its initially disordered structure, consistent with the weak and poorly defined anodic response. With increasing time, the metastable LCO surface gradually rearranges from an HT- to an LT-dominated structure, accompanied by increased Li/Co cation mixing. The partial occupation of Li-layer sites by Co^3+^ can obstruct Li^+^ diffusion pathways and induce local Li^+^ trapping, thereby weakening reversible Li^+^ (de)intercalation and increasing interfacial polarization.^48,49^ A weak Co_3_O_4_-related feature at ∼520 cm^−1^ is already present in the LCO film, reflecting the sensitivity of multicomponent-target sputtering to deposition-process design.^50^ After 24 days, a new prominent peak appears at ∼690 cm^−1^ and continuously intensifies with time, which can be assigned to the A_1g_ mode of Co_3_O_4_.^51^ Together with the Li_2_CO_3_ passivation layer, this structural transformation and surface oxidation impede Li^+^ transport, accounting for the attenuation of the anodic peak and the continuous negative shift of the cathodic peak. These changes are most pronounced in R3, consistent with its earlier and more severe electrochemical degradation.

XPS measurements further corroborate the progressive chemical reconstruction of the LCO surface (Fig. S10).^52^ The increasing carbonate contribution, attenuation of the lattice-Li signal, and binding-energy evolution support the growth of a Li_2_CO_3_-rich passivation layer and concurrent near-surface oxidation. These changes are most pronounced in R3, where the C 1s (CO_3_^2−^) component exhibits the largest binding-energy shift (−0.64 eV), confirming more extensive local chemical degradation. After 44 days of exposure, the Li 1s binding energies of the three domains converge to ∼55.5 eV, indicating the development of a similarly passivated surface state across the film.

The correlative structural and chemical analyses reveal that morphological changes and chemical reconstruction influence different aspects of the electrochemical response. Overall, the interfacial chemical effects, including interfacial byproduct formation and phase transformation, introduce a dominant chemical contribution to the electrochemical response by reducing Li^+^ transport kinetics. To quantitatively distinguish their individual roles, finite-element simulations were subsequently performed by independently introducing morphological and chemical descriptors into the electrochemical model.

### Quantitative decoupling of morphological and chemical contributions through multiphysics modelling

The correlative electrochemical, structural, and spectroscopic analyses collectively demonstrate that surface morphological changes and interfacial chemical reconstruction occur simultaneously. However, because both processes continuously influence the measured electrochemical response, their respective contributions cannot be quantitatively distinguished from experimental observations alone. To resolve this challenge, finite-element simulations were performed to independently evaluate the effects of surface morphology and interfacial chemistry by introducing two physically meaningful descriptors: the effective roughness factor, *a*_F_, which accounts for morphological changes of the electrode surface, and the effective lithium-ion diffusion coefficient, *D*_s_, which reflects chemically induced transport limitations.

To account quantitatively for the combined effects of surface roughening and crack propagation, we defined *a*_F_ using the following relationship:

1 *a*_F_ = *a*_RF_ + *a*_crack_ = *a*_RF_ + 50 × (1 − e^−0.01*t*_days_^)

where *a*_F_ is the contact area ratio obtained from AFM height information, which is calculated as the ratio between the actual contact area under the pipette meniscus and the theoretical morphology area. *a*_crack_ is a crack correction factor introduced to quantitatively account for the effect of time-dependent crack propagation.

To capture the progressive loss of Li^+^ mobility caused by Li_2_CO_3_ accumulation, Co_3_O_4_ enrichment, and the HT-to-LT phase evolution, we used a time-dependent interpolation between the initial and aged states to represent the effective diffusion coefficient.2
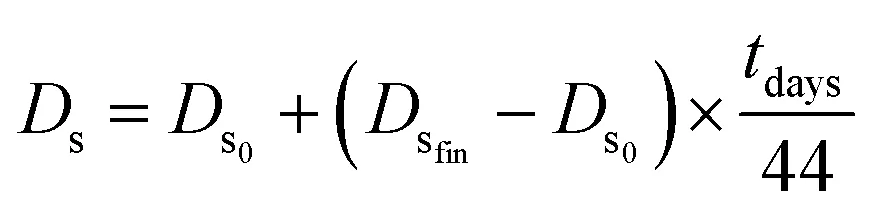
where *D*_s_0__ and *D*_s_fin__ are the effective Li^+^ diffusion coefficients in LCO on day 0 and day 44, respectively, and *t*_days_ is the air-exposure time in days. The two descriptors were implemented in a 2D axisymmetric SECCM-LCO finite-element model that couples the Tertiary Current Distribution in the electrolyte with the Transport of Diluted Species in LCO. Interfacial charge transfer was described by Butler–Volmer kinetics. The model geometry, governing equations, variable definitions, boundary conditions, and parameter values are provided in Fig. S11 and Tables S1–S4 of the SI. Based on this model, the day 0 condition is taken as a representative example to illustrate the concentration field response during the anodic and cathodic scanning processes (Fig. S12), to clarify the variations of Li^+^ transport near the interface and mass transfer on the solution side across different potential ranges.

The simulations indicate that during the anodic process, the interfacial reaction driving force increases with increasing potential, leading to enhanced migration of Li^+^ from the electrode side toward the interface, corresponding to the deintercalation process. In contrast, during the cathodic process, as the potential decreases, Li^+^ more readily undergoes intercalation into LCO, resulting in an initial increase in Li^+^ concentration near the interface. When the potential deviates from the peak potential, solid-phase diffusion gradually becomes the rate-limiting step, and the interfacial concentration variation correspondingly slows down. On this basis, the cathodic peak potential at day 0, 3.67 V, is selected as a fixed potential to compare the interfacial concentration responses at different exposure times (Fig. 5a), revealing that the Li content in the vicinity of the LCO interface increases over time, which indicates an enhanced tendency for local Li accumulation on the solid phase side. This behaviour arises from the continuous decrease in the solid-state diffusion coefficient *D*_s_ with exposure time, which is attributed to the gradual coverage of the interface by byproducts that increase transport resistance, together with the filling of cracks and pores that reduces the number of effective migration pathways, thereby promoting Li^+^ accumulation near the interface. Considering that the cathodic peak potential continuously shifts to more negative values with increasing exposure time in the experiments, the concentration response at each time is further extracted at the respective cathodic peak potentials (Fig. 5b). At these peak potentials, both the Li^+^ concentration inside the pipette and the Li^+^ concentration in the LCO electrode increase over time, indicating that structural transformation of LCO requires a stronger potential driving force to sustain an intercalation rate, which is consistent with the experimentally observed negative shift of peak potentials and the enhancement of interfacial polarization in SECCM measurements.

**Fig. 5:**
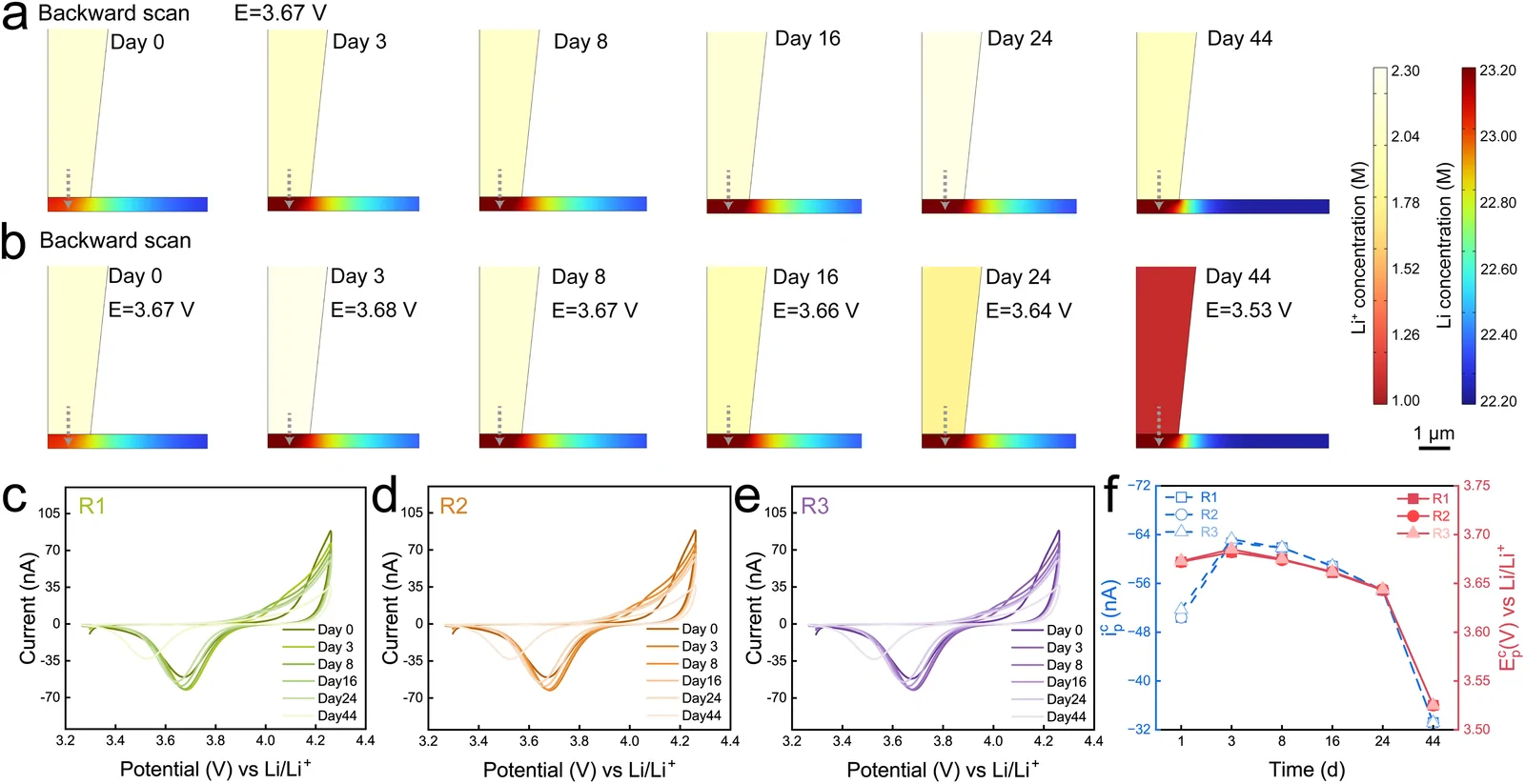
Simulation analysis of the spatiotemporal evolution of electrochemical kinetics in LCO domains. (a) Concentration fields of Li in the LCO and Li^+^ in the probe solution near the LCO interface for the R1 domain at 3.67 V *vs.* Li/Li^+^ under different air-exposure times (day 0, 3, 8, 16, 24, and 44). (b) Concentration fields of Li in the LCO and Li^+^ in the probe solution near the LCO interface for the R1 domain at the cathodic peak potential at different exposure times (day 0, 3, 8, 16, and 24). CV curves of the (c) R1, (d) R2, and (e) R3 domains at different exposure times under the combined control of *D*_s_ and *a*_F_. (f) Line plots of the simulated cathodic peak potential and peak current corresponding to the R1, R2, and R3 domains as a function of air-exposure time.

Fig. 5c–e presents the simulated CVs of the R1, R2, and R3 microdomains at extended exposure durations. With increasing exposure time, the cathodic peak exhibits a continuous negative shift, while the anodic peak progressively weakens and becomes less discernible after day 16, in good agreement with the trend observed in the SECCM measurements. To distinguish the independent roles of *D*_s_ and *a*_F_ in governing the electrochemical behaviour, simulations were further performed by allowing only one parameter to evolve while keeping the other constant (Fig. S13). The results show that *D*_s_ and *a*_F_ exert fundamentally different influences on the voltammetric features. Variation of *D*_s_ alone is sufficient to induce a clear negative shift of the peak potential, whereas variation of *a*_F_ primarily modulates the peak current magnitude and the gradual attenuation of the anodic peak. These observations indicate that the decline in transport capability caused by changes in interfacial chemical properties is the key factor driving the peak potential shift, while morphological changes mainly affect the current magnitude through their impact on the effective contact area. The line plots of cathodic peak potentials and peak currents extracted from the simulated CV (Fig. 5f and S14) show that, when *D*_s_ and *a*_F_ evolve simultaneously, the simulated trends of peak potential and current are in close agreement with the experimental trend in Fig. 2e. This result is consistent with the conclusions drawn from AFM and Raman measurements, in which surface roughening and crack evolution introduce morphology effects that primarily determine the effective reaction area and the current magnitude, whereas byproduct deposition and phase transformation introduce interfacial chemical effects that dominate the limitation of Li^+^ transport and the negative shift of peak potential. Overall, the simulations bridge the gap between localized electrochemical observations and interfacial physicochemical reconstruction by quantitatively distinguishing the respective roles of surface morphology and interfacial chemistry in determining electrochemical behaviour.

## Conclusions

In summary, this work presents a multimodal analysis strategy to interrogate the spatiotemporal evolution of localized electrochemical behaviour during the LCO thin film evolution process. Correlative electrochemical and physicochemical analyses reveal that surface degradation proceeds through highly heterogeneous pathways despite the initially uniform structure of the films. The combined experimental and theoretical results demonstrate that surface morphological changes and interfacial chemical reconstruction govern different aspects of the electrochemical response, where surface morphology primarily regulates electrochemical accessibility and therefore dominates the evolution of localized current magnitude, whereas interfacial chemistry progressively limits lithium-ion transport kinetics, leading to the continuous cathodic peak shift observed during degradation. By independently introducing the roughness factor and the effective lithium-ion diffusion coefficient into the multiphysics model, the respective contributions of morphological and chemical reconstruction can be quantitatively distinguished. This decoupling strategy provides a mechanistic interpretation of the seemingly contradictory evolution of current magnitude and reaction polarization during surface reconstruction, while highlighting the complementary roles of localized electrochemical measurements and correlative structural characterization. Although demonstrated here using LCO thin films as a model system, the conceptual approach developed in this work offers a practical strategy for investigating heterogeneous electrochemical interfaces where structural reconstruction and chemical reconstruction occur simultaneously. Future advances in meniscus confinement, low-current detection, and high-precision positioning may enable smaller-diameter nanopipettes to extend this correlative strategy to individual particles and intraparticle nanoscale heterogeneities. These findings not only advance the understanding of surface degradation in existing battery cathodes but also provide a foundation for future studies aimed at quantitatively correlating local interfacial evolution with electrochemical function in complex energy-storage materials.

## Author contributions

Yuxia Guan: conceptualization, methodology, investigation, formal analysis, data curation, visualization, writing – original draft. Pingshi Wang, Ning Gao, and Penghui Liu: investigation, formal analysis. Weiwei Cao and Shujiang Ding: resources, investigation. Lingjie Meng and Kai Xi: supervision, writing – review & editing. Rui Gao: conceptualization, supervision, writing – review & editing.

## Conflicts of interest

There are no conflicts to declare.

## Supplementary Material

SC-017-D6SC06380B-s001

## Data Availability

The data supporting this article have been included as part of the supplementary information (SI). Supplementary information is available. See DOI: https://doi.org/10.1039/d6sc06380b.
