# Supplementary material for: Decoupling the effects of surface morphology and interfacial chemistry on localized electrochemical kinetics in LiCoO2 cathodes
Source: Chem Sci. 2026 Sep 9;17(42):20545–54. doi: 10.1039/d6sc06380b (PMC13583917; doi:10.1039/d6sc06380b)
Supplement: Supplementary file 1 [file SC-017-D6SC06380B-s001.pdf]

Supplementary Information (SI) for Chemical Science.
This journal is © The Royal Society of Chemistry 2026

Supporting Information

Decoupling the effects of surface morphology and interfacial chemistry on localized electrochemical kinetics in $LiCoO_2$ Cathodes

Yuxia Guan [a], Pingshi Wang [a], Ning Gao [a], Penghui Liu [a,b], Weiwei Cao [c], Shujiang Ding [a,d],

*Lingjie Meng *[a,e,f], Kai Xi *[a,d], and Rui Gao *[a,d,f]*

a. *School of Chemistry, Xi'an Jiaotong University, Xi'an 710049, China*

b. *School of Future Technology, Xi'an Jiaotong University, Xi'an 710049, China*

c. *Xi'an Institute of Optics and Precision Mechanics, Chinese Academy of Sciences, Xi'an, 710119, China*

d. *Engineering Research Center of Energy Storage Materials and Devices, Ministry of Education, National Innovation Platform (Center) for Industry-Education Integration of Energy Storage Technology, Xi'an Jiaotong University, Xi'an, 710049, China*

e. *Instrumental Analysis Center of Xi'an Jiaotong University, Xi'an 710049, China.*

f. *Ningxia Advanced Intelligent Sensing and Control Technology Innovation Team & Ningxia Key Laboratory of Bioimaging and Intelligent Diagnostics, North Minzu University, Yinchuan, 750021, China*

**E-mail: rui.gao@xjtu.edu.cn, menglingjie@mail.xjtu.edu.cn, kx210.cam@xjtu.edu.cn*

Table of contents

Experimental section 3

S1. Fabrication and structural characterization of the LCO film 6

S2. Measurement of electrochemical spatiotemporal heterogeneity in different microdomains of the LCO film 9

S3. Dynamic contact angle measurements of the R3 microdomain on the LCO film .14

S4. Measurement of the spatiotemporal heterogeneity of surface morphology in different microdomains of the LCO film 15

S5. XPS analysis of different microdomains on the LCO film on Day 0 and Day 44.17

S6. Multiphysics simulation analysis of electrochemical kinetics in different domains of the LCO film. 19

References 29

Experimental section

Chemical Reagents and Materials.

Lithium chloride (LiCl, 99.0%, Aladdin), lithium cobalt oxide ($LiCoO_2$, 99.9%, ZhongNuo Advanced Material), platinum (Pt, 99.9%, ZhongNuo Advanced Material), and titanium (Ti, 99.9%, ZhongNuo Advanced Material) targets, along with single-side polished silicon (100) wafers (Kaihua Shunchen Electronic Technology), were used as received.

Thin Film Preparation.

LCO thin films were prepared using a high-vacuum magnetron sputtering deposition system (TRP450, Sky Technology Development Co., Ltd.). Square silicon wafers (1 cm^2) were used as substrates. Prior to LCO deposition, Ti and Pt layers were sequentially deposited onto the Si wafers by direct current magnetron sputtering in a vacuum chamber under an Ar atmosphere. The Ti layer was employed to enhance adhesion, while the Pt overlayer served as a current collector and a conductive barrier to prevent Ti diffusion. Subsequently, the LCO film was fabricated via radio frequency magnetron sputtering in an Ar/O_2 (3:1) mixture. A 5 min pre-sputtering step was performed to clean the target surface. During deposition, the substrate was rotated at 10 rpm to ensure film uniformity. The RF sputtering power was set to 140 W, the working pressure was maintained at 5 mTorr, and the target-to-substrate distance was 5 cm. The deposition time was 2 h. The as-deposited films were then exposed to ambient air for extended periods, and samples were collected on days 0, 3, 8, 16, 24, and 44 for subsequent measurements.

Fabrication of nanopipette.

Single-barrel nanopipette with a 4 µm aperture was fabricated from borosilicate glass capillaries (BF-100-50-7.5, Sutter Instrument, U.S.A.) using a CO_2 laser puller (P-2000, Sutter Instrument, U.S.A.). A two-step pulling program was employed. The parameters for the first step were: HEAT 350, FIL 3, VEL 10, DEL 250; and for the second step: HEAT 350, FIL 3, VEL 10, DEL 220, PUL 100. The tip diameter was measured using a field-emission scanning electron microscope (Gemini SEM 500,

ZEISS) operating at an acceleration voltage of 5 keV. As shown in Figure S4a, the resulting tip diameter was confirmed to be 4 μm.

Electrochemical Measurements.

The LCO film was mounted on the sample stage of a scanning electrochemical cell microscope (SECCM, ElProscan ELP3, HEKA ElMEA) and connected as the working electrode. A nanopipette filled with 1 M LiCl aqueous solution was fixed on the holder. An Ag/AgCl wire was inserted into the pipette to serve as the quasi-reference counter electrode (QRCE) and connected to the current amplifier of the SECCM. The nanopipette was approached to the sample surface using a stepper motor, followed by fine positioning under piezoelectric control until meniscus contact was established. Upon surface detection, localized cyclic voltammetry (CV) was performed for 20 cycles on specific microdomains (designated as R1, R2, and R3) of the LCO films after different exposure times (days 0, 3, 8, 16, 24, and 44). The CV measurements were conducted in a potential window of -1 V to 1 V (vs. Ag/AgCl) at a scan rate of 100 mV/s. Subsequently, high-resolution electrochemical imaging was performed in the corresponding domains using the hopping mode, with an image resolution of 5 × 5 pixels and a step size of 10 μm.

AFM Roughness Imaging.

The surface roughness of the samples was evaluated using an atomic force microscope (SPM-9700HT, Shimadzu). Measurements were conducted on 5 × 5 μm^2 areas within the specific microdomains (R1, R2, and R3) of the LCO films. Two-dimensional (2D) and three-dimensional (3D) topographical images were acquired at a scan rate of 1 Hz with a resolution of 512 lines per frame, and subsequently processed using SPM Manager software. Key roughness parameters, including the arithmetic mean roughness (Ra), root-mean-square roughness (Rq), and ten-point mean height (Rz), were extracted. For each parameter, the overall average value, the areal average from a randomly selected 2 × 2 μm^2 sub-domain, and the linear average along a 5 μm profile line were calculated. These measurements and parameter extractions were systematically performed on the three designated microregions of the films stored for

different periods (days 0, 3, 8, 16, 24, and 44).

Raman Surface Structure and Composition Measurement.

The surface structure and composition of the samples were analysed using a Raman spectrometer (InVia Qontor, Renishaw) equipped with a 532 nm excitation laser. The laser was operated at 10% of its maximum output, corresponding to a nominal power of 5 mW, 10 s accumulation time, and three accumulations. Full Raman spectra were acquired from the specific microdomains (R1, R2, and R3) of the LCO films after different exposure times (days 0, 3, 8, 16, 24, and 44). The acquired spectral data were then carefully processed, with a specific focus on the 400~700 cm^{-1} frequency range. Peak deconvolution and fitting were performed in this domain to quantitatively analyse the structural evolution of the films.

Material characterization.

Film thickness was characterized using a field-emission scanning electron microscope (Gemini SEM 500, ZEISS). The wettability of the LCO films on days 0 and 44 was evaluated using a drop shape analyser (DSA100S, KRUSS). Specifically, dynamic contact angle measurements were performed on the R3 microdomain, with the contact angle values extracted at exactly 5 s after a droplet of the 1 M LiCl aqueous probe liquid contacted the surface. Furthermore, X-ray photoelectron spectroscopy (PHI GENESIS 500, ULVAC-PHI) was employed to elucidate the surface chemical compositions and bonding configurations of the samples. The XPS analyses were conducted under ultrahigh vacuum conditions (10^{-10} mbar) using Al Kα radiation, with photoelectron emission angles set at 15° and 90° relative to the sample surface.

Finite Element Simulations.

Finite element method (FEM) simulations of the nanoelectrochemical cell at the tip of the SECCM probe were implemented and solved using MATLAB software (MATLAB R20226a, The MathWorks, Inc., Natick, MA, USA).

S1. Fabrication and structural characterization of the LCO film

SEM images of different microdomains on the LCO film at different exposure times

The LCO films in this work were prepared on Si substrates by magnetron sputtering. Due to the high-energy plasma environment and oxygen-rich conditions during sputtering, LCO easily reacts with the Si substrate to form alloys. [1] Therefore, in this study, a 20 nm thick Ti layer was first deposited on the Si substrate as an adhesion layer by direct current magnetron sputtering, followed by a 50 nm Pt layer to block interfacial reactions and enhance conductivity. Finally, a uniform LCO film was deposited on the Pt layer by radio frequency magnetron sputtering at 140 W for 2 h. SEM and energy-dispersive X-ray spectroscopy (EDS) elemental mapping results show that the LCO film has a dense surface, consisting of closely packed nanograins with sizes ranging from 30 to 50 nm (Figure S1a and b). The corresponding particle size distribution histogram shows that the average particle size of the LCO film is 39±9 nm. Cross-sectional SEM images indicate that the film thickness is highly uniform, with a deposition rate of 3.33 nm/min. EDS mapping results further confirm the layered distribution of Co, O, Pt, Ti, and Si elements (Figure S1c). The thickness of the LCO layer is ~400 nm (Figure S1c).

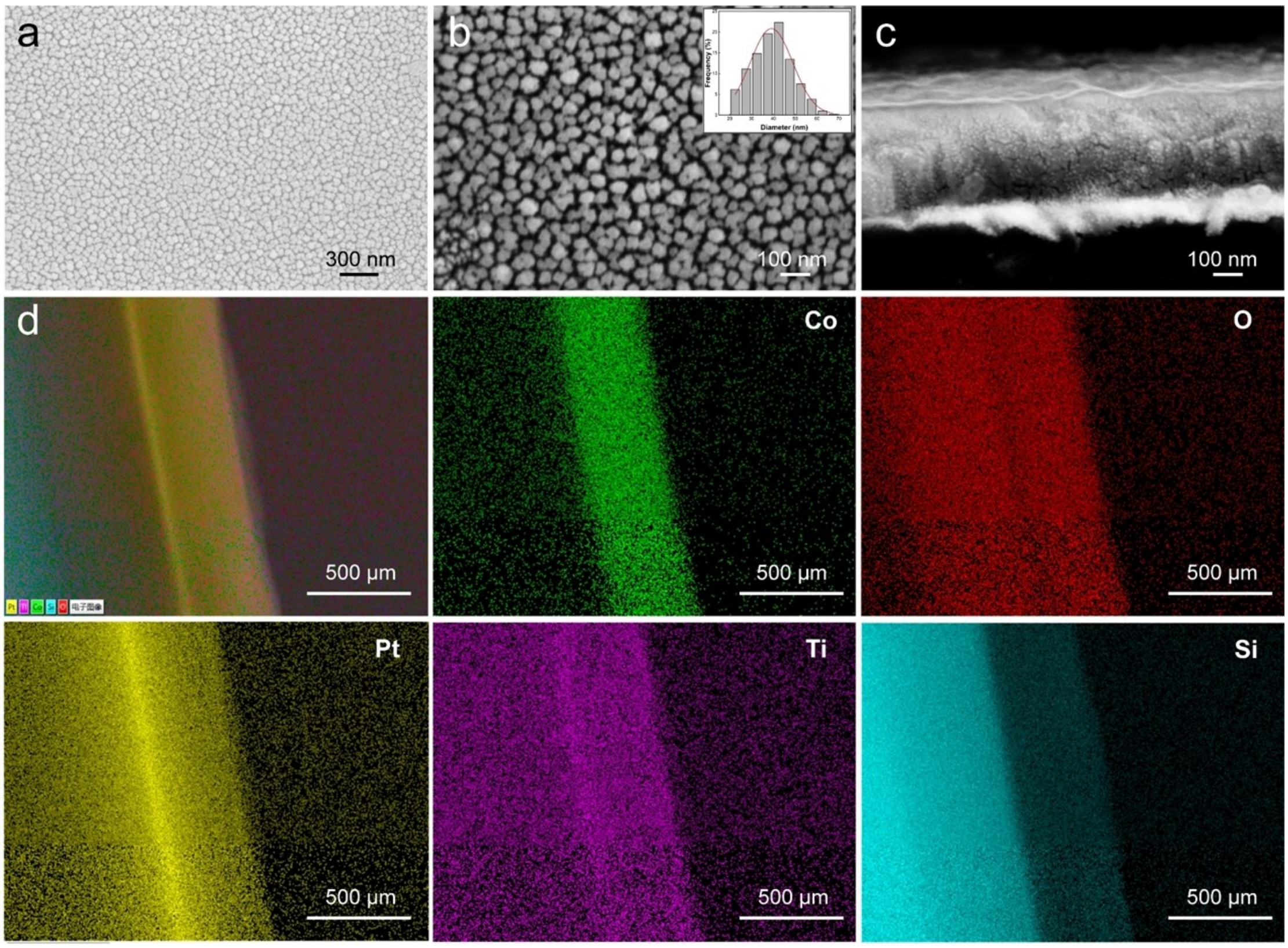

Figure S1. (a) Low-magnification top-view SEM image of the freshly prepared magnetron-sputtered LCO film on a Pt/Ti/Si substrate. (b) High-magnification top-view SEM image of the LCO film and the corresponding particle size distribution histogram. (c) Cross-sectional SEM image of the LCO film. (d) Corresponding cross-sectional EDS mapping images of Co, O, Pt, Ti, and Si elements.

Macroscopic surface evolution of the LCO film exposed to ambient air for 44 days.

The LCO film was exposed to ambient air for 44 days. It was observed that starting from Day 3, the film surface began to exhibit obvious macroscopic heterogeneous evolution. Based on the distinct surface evolution features, the film could be roughly divided into three domains R1, R2, and R3, from the central domain to the edge of the film. This regional heterogeneity became increasingly pronounced with prolonged exposure time.

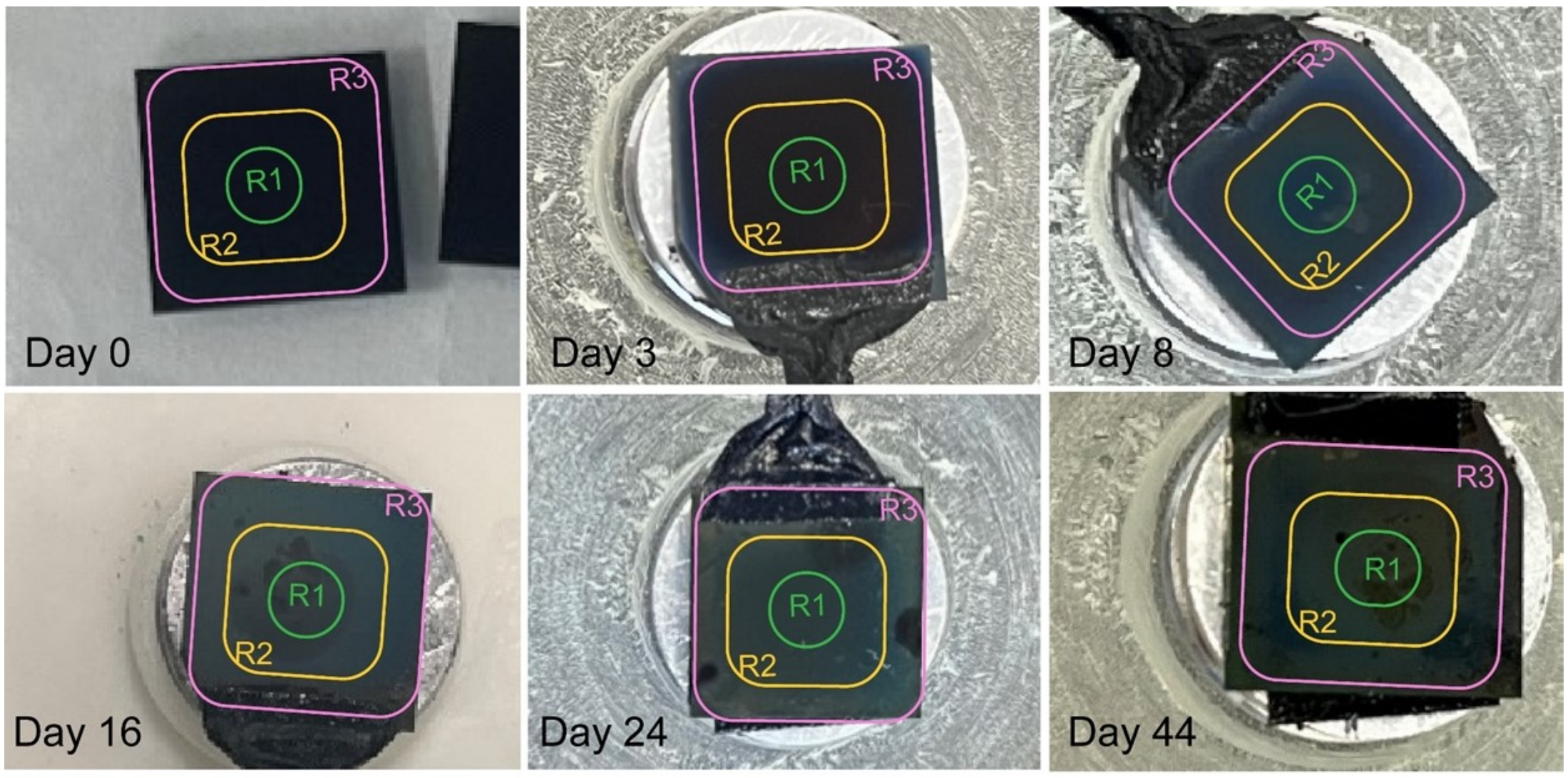

Figure S2. Evolution of the macroscopic domain partitioning (domains R1, R2, and R3) on the LCO thin film surface after 0, 3, 8, 16, 24, and 44 days of exposure to ambient air.

S2. Measurement of electrochemical spatiotemporal heterogeneity in different microdomains of the LCO film

Characterization of nanopipette

In this work, a single-barrel pipette with an aperture diameter of ~4 μm was employed as the probe for SECCM measurements.

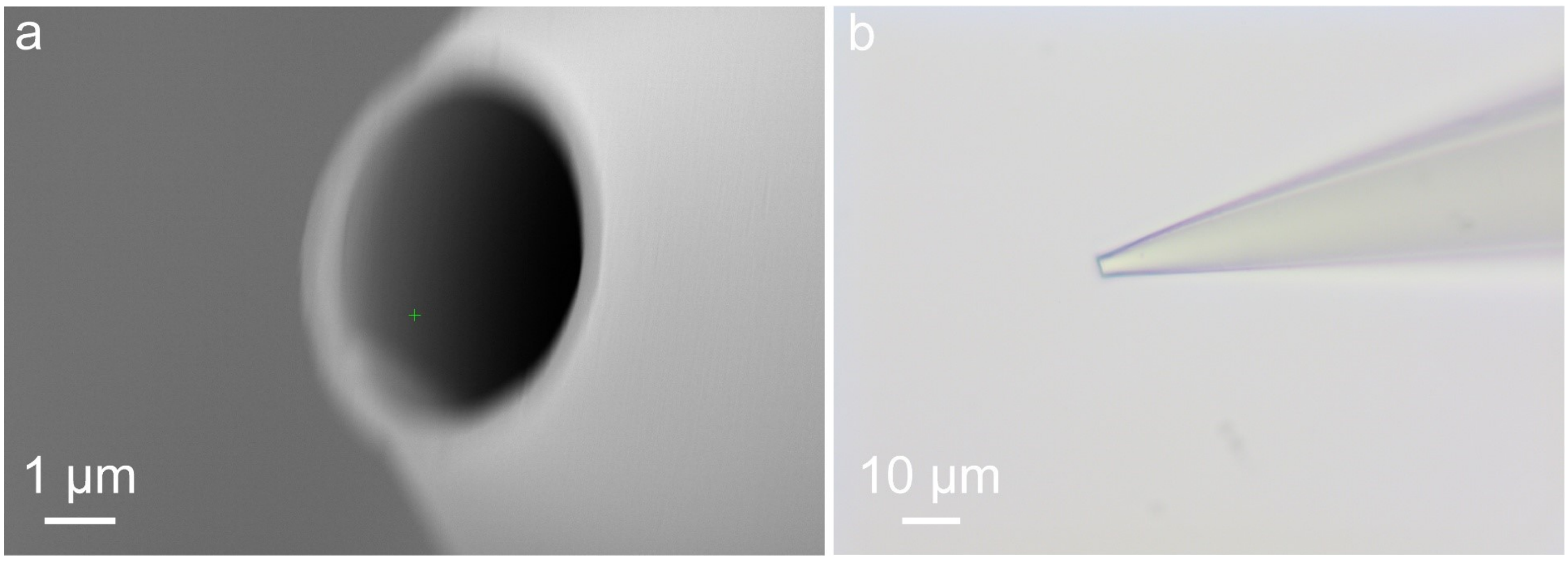

Figure S3. (a) SEM image of the pipette aperture with a diameter of ~4 μm (the tip was sputter-coated with gold prior to imaging). (b) Side-view optical image of the corresponding pipette.

SECCM Setup

The SECCM instrumentation and operating configuration are shown in Figure S4. The system consisted of an EIProScan ELP3 electrochemical probe scanner (HEKA EIMEA), a bipotentiostat model, a computer-controlled positioning system, and a side-view optical microscope (Figure S4a). The nanopipette-positioning assembly and sample stage were mounted on an antivibration table, and the system was operated inside a Faraday cage to minimize mechanical vibration and electrical noise. A schematic of the principal components and their connections is presented in Figure S4b.

SECCM operates by confining the electrolyte within the meniscus formed at the nanopipette tip. When the meniscus contacts the conductive LCO substrate, a localized electrochemical cell is established over an area defined by the meniscus footprint. As illustrated in Figure S4c, the LCO substrate serves as the working electrode, while the Ag/AgCl wire inside the nanopipette functions as the QRCE. The nanopipette is positioned above the selected region using the mechanical positioning system and then precisely controlled in the vertical direction by the z-piezoelectric positioner. Meniscus contact with the substrate closes the electrical circuit and generates a measurable current response, which is used to detect surface contact. The localized electrochemical response is subsequently recorded through the bipotentiostat model. In hopping mode, the nanopipette is repeatedly approached, contacted, retracted, and translated to the next predefined position, enabling continuous electrochemical mapping across multiple locations within the selected region.

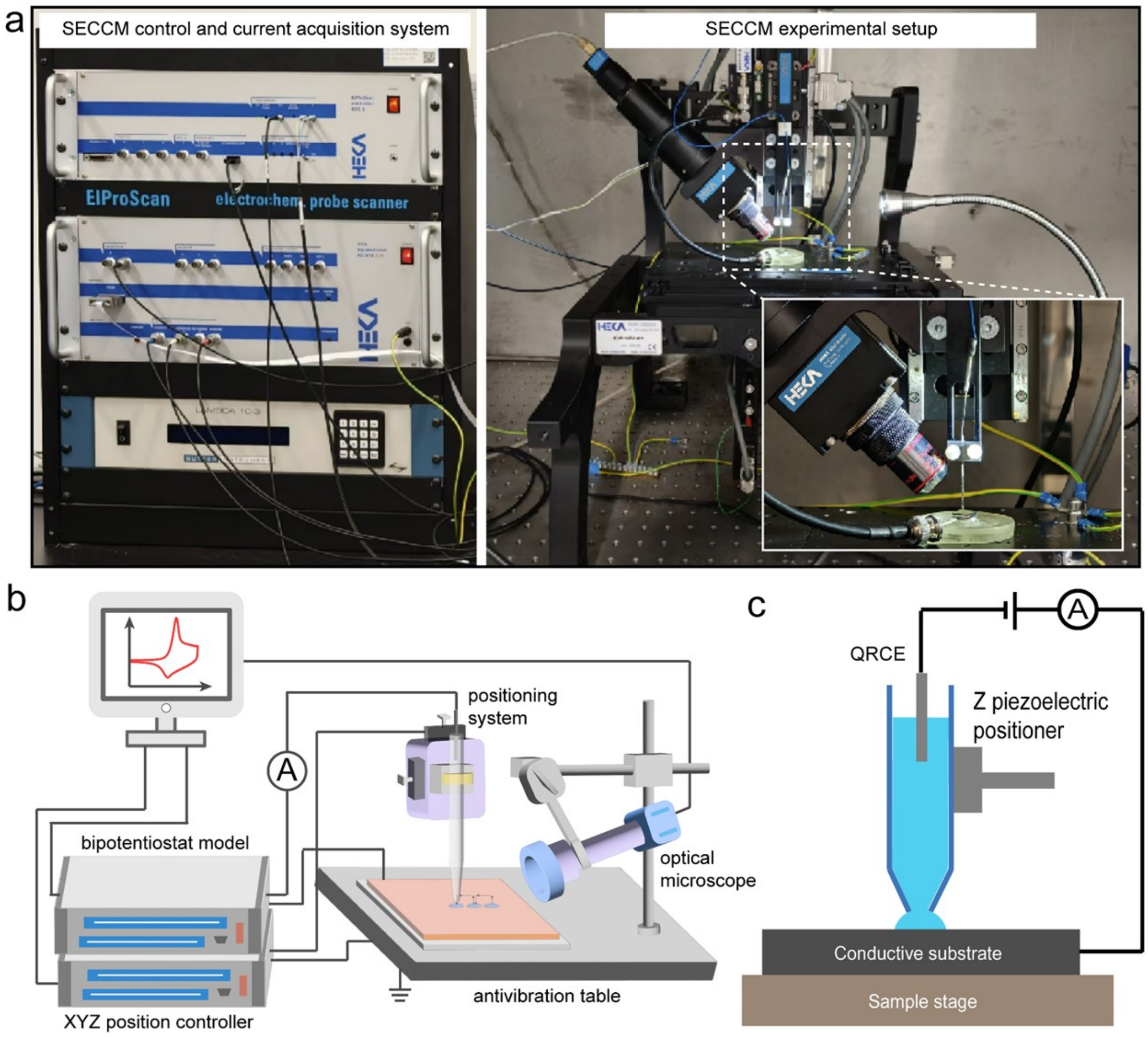

Figure S4. SECCM setup. (a) Photographs of the control system and experimental apparatus. (b) Schematic of the SECCM instrumentation. (c) Two-electrode configuration of the localized electrochemical cell.

CV curves of different microdomains on the LCO film at various exposure times

Figure S5 presents the second-cycle CV curves of the three distinct microdomains (R1, R2, and R3) on the LCO film at various exposure times. These CV curves correspond to the extracted peak current and peak potential plots shown in Figure 2e of the main text. The first CV cycle is omitted here, as it primarily serves as an electrochemical activation step for the LCO electrode.

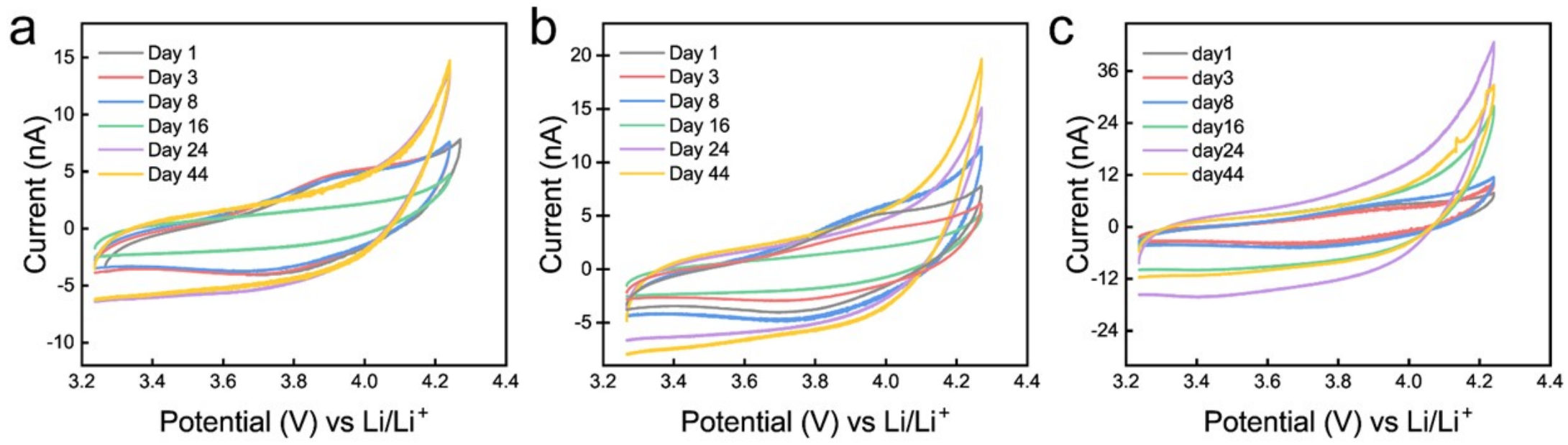

Figure S5. Second-cycle CV curves of the (a) R1, (b) R2, and (c) R3 microdomains on the LCO film after exposure to ambient air for 1, 3, 8, 16, 24, and 44 days. The SECCM measurements were performed using a single-barrel micropipette (~4 μm in diameter) filled with 1 M LiCl solution at a scan rate of 100 mV/s.

Cycling stability measurements of different microdomains on the LCO film at different exposure times

Figure S56shows the CV curves of the R1, R2, and R3 microdomains on the LCO film over 20 cycles at various exposure times. For clarity, only the 2nd, 5th, 10th, 15th, and 20th cycles are plotted. To evaluate the cycling stability, the cathodic peak current of the 2nd cycle is defined as the initial reference value ($i^{c}_{p,0}$). The 2nd cycle, rather than the 1st cycle, was selected as the reference because the initial cycle is typically accompanied by an electrochemical activation process, and using the 2nd cycle as the baseline minimizes the influence of this initial activation on the subsequent comparison. The corresponding peak currents for the subsequent *n*-th cycles (5th, 10th, 15th, and 20th) are denoted as $i^{c}_{p,n}$. The retention efficiency is then calculated using the ratio of $i^{c}_{p,n}/i^{c}_{p,0}$, which corresponds to the retention efficiency plots for the R1, R2, and R3 microdomains presented in Figures 2a-c in the main text.

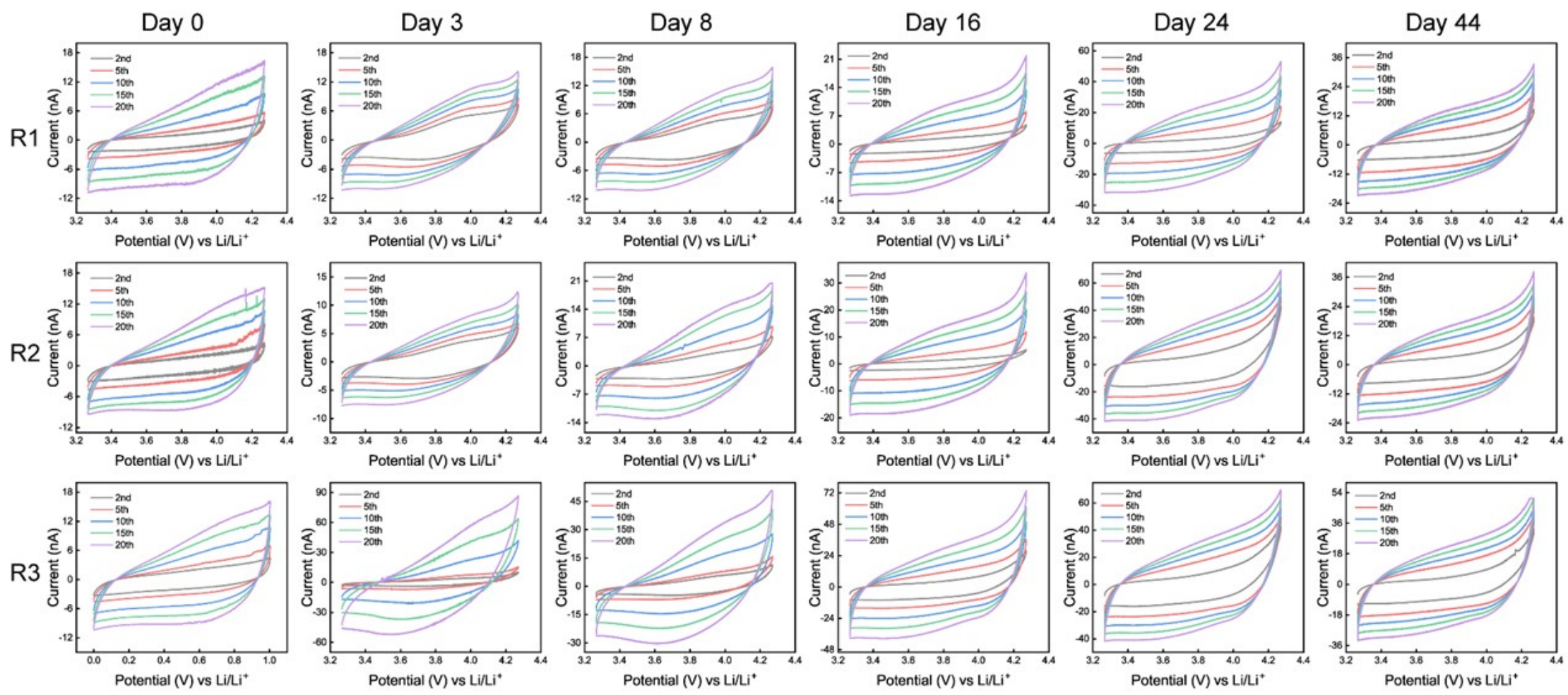

Figure S6. Cycling stability evaluated by continuous CV measurements of the R1, R2, and R3 microdomains on the LCO film at the indicated exposure times. Selected CV curves (the 2nd, 5th, 10th, 15th, and 20th cycles) are displayed. The scan rate is 100 mV/s.

S3. Dynamic contact angle measurements of the R3 microdomain on the LCO film

Dynamic contact angle measurements were performed by dispensing a droplet of 1 M LiCl aqueous solution onto the R3 microdomain. The contact angle evolution was continuously recorded over a 5-second period. The substantial increase in the contact angle from Day 0 to Day 44 clearly demonstrates a significant decrease in surface hydrophilicity induced by prolonged exposure to ambient air.

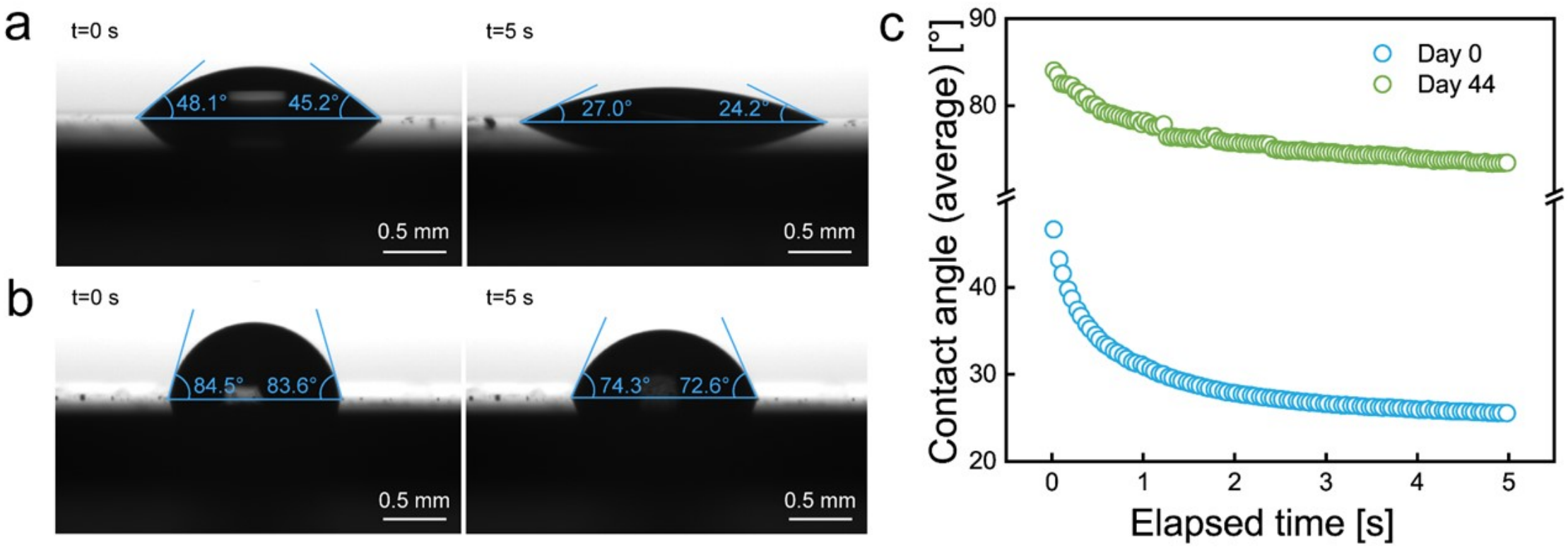

Figure S7. (a) Optical images showing the contact angles of a 1 M LiCl droplet on the R3 microdomain at 0 s and 5 s on Day 0. (b) Optical images showing the contact angles on the R3 microdomain at 0 s and 5 s on Day 44. (c) Dynamic contact angle evolution profiles for the R3 microdomain over a 5 s period on Day 0 and Day 44.

S4. Measurement of the spatiotemporal heterogeneity of surface morphology in different microdomains of the LCO film

3D AFM images of different microdomains on the LCO film at different exposure times

Figure S8 presents the 3D AFM topographic images corresponding to the 2D maps shown in Figure 3b of the main text. It clearly illustrates the topographic evolution of the LCO surface. With increasing exposure time to ambient air, continuous growth and agglomeration of surface particles can be observed across all microdomains, which eventually evolve into a relatively stabilized morphological state.

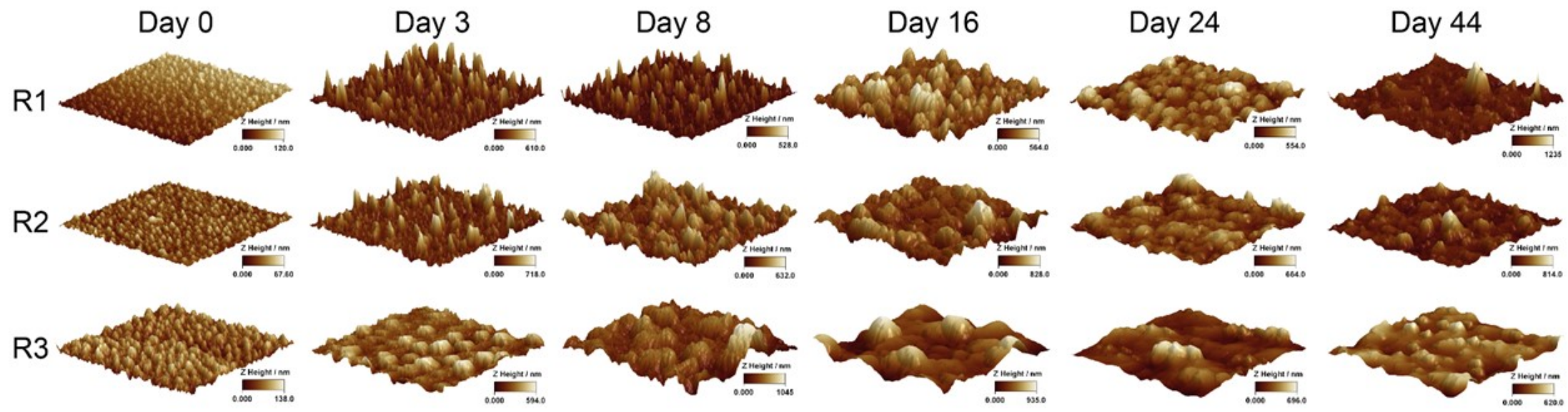

Figure S8. 3D AFM topographic images of the R1, R2, and R3 microdomains on the LCO film after exposure to ambient air for 0 to 44 days. The scan area is 5×5 μm^2.

Sub-regional average roughness parameters of different microdomains on the LCO film at different exposure times

Due to the continuous particle growth and agglomeration, the LCO surface exhibits significant spatial heterogeneity, leading to localized roughness fluctuations across the 5×5 μm^2 AFM scan area. To minimize the influence of these localized anomalies and ensure statistical reliability, sub-regional sampling was performed. We randomly selected three 2×2 μm^2 sub-regions within each mapped area to calculate the average roughness parameters (Ra, Rq, and Rz). As shown in Figure S9, the evolutionary trends of these sub-regional averages are highly consistent with the overall areal averages presented in Figures 3c-e of the main text.

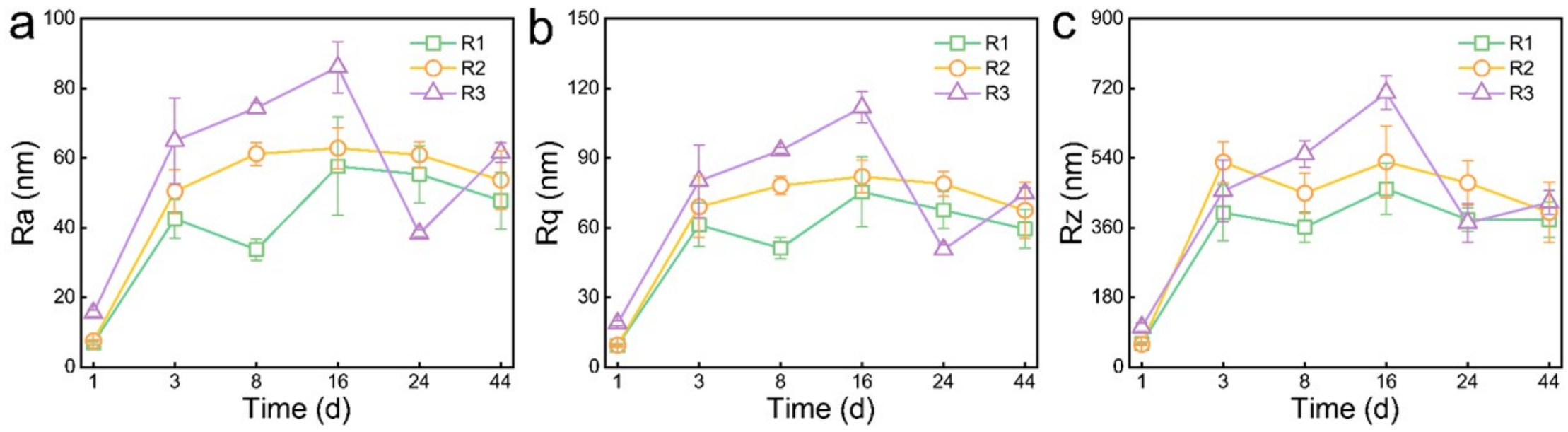

Figure S9. (a) Ra, (b) Rq, and (c) Rz sub-regional average values of the R1, R2, and R3 microdomains on the LCO film at different exposure times. The data points are averaged from three randomly selected 2×2 μm^2 sub-regions, with error bars representing the standard deviations.

S5. XPS analysis of different microdomains on the LCO film on Day 0 and Day 44

To investigate the evolution of the LCO surface chemistry after prolonged air exposure, high-resolution XPS spectra of C 1s, Li 1s, and O 1s were acquired (Figure S10). For the as-deposited film, the C 1s spectrum confirms the presence of surface carbonate (CO_3^{2-}) species and C–O bonds. The Li 1s spectrum is dominated by contributions from Li_2CO_3/LiOH, together with a distinct lattice-Li component from the underlying LCO. Because the LCO surface is covered by a spontaneously formed Li-rich passivation layer, the Co 3p signal overlapping with the Li 1s region is comparatively weak. Consistently, the O 1s spectrum can be resolved into surface oxygen associated with Li_2CO_3/LiOH and lattice oxygen associated with Co–O bonds in LCO.

Upon air exposure, reactions involving the LCO surface, H_2O, and CO_2 promote the formation of surface hydroxyl species and the continued accumulation of Li_2CO_3.[2] After 44 days, pronounced binding-energy shifts are observed in the C 1s, Li 1s, and O 1s spectra. [3] The growing Li_2CO_3-rich layer progressively attenuates the lattice-Li signal, which becomes undetectable by Day 44, while the carbonate-related components in the C 1s and Li 1s spectra shift to lower binding energies. In contrast, the lattice-oxygen and surface-oxygen components in the O 1s spectra shift to higher binding energies, consistent with the increasing contribution of carbonate/hydroxyl species and the oxidation of the near-surface LCO layer. These spectral changes corroborate the Raman results, confirming the progressive development of a poorly Li^+-conducting surface passivation layer during air exposure.

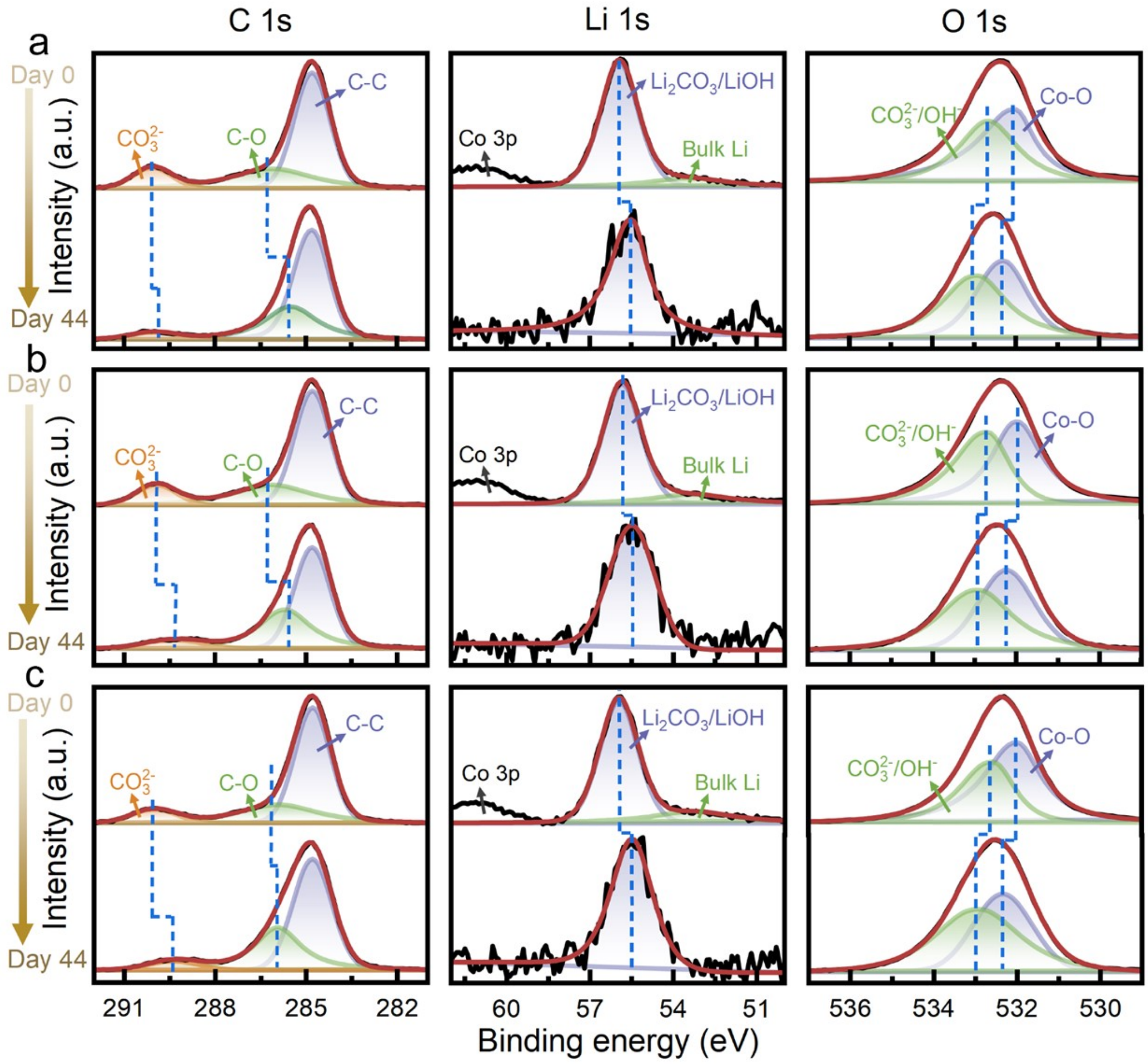

Figure S10. High-resolution XPS spectra and peak fitting of the C 1s, Li 1s, and O 1s for the (a) R1, (b) R2, and (c) R3 microdomains on the LCO film on Day 0 and Day 44.

S6. Multiphysics simulation analysis of electrochemical kinetics in different domains of the LCO film.

Geometric model and schematic of the finite element model for SECCM measurements on the LCO surface

To establish a quantitative link between experimentally observed structural evolution and localized electrochemical responses, a 2D axisymmetric finite-element model was developed. A quadrilateral with a base length of 1.5 μm and a height of 25 μm represented one half of the axisymmetric pipette, and a 400 nm-wide rectangle represented the LCO electrode. The electrolyte domain was governed by the Tertiary Current Distribution interface to describe ionic transport and potential distribution, whereas the solid LCO domain was governed by the Transport of Diluted Species interface to describe solid-state Li^+ diffusion. The two domains were coupled at the electrolyte-LCO interface.

At the $LiCoO_2$/LiCl interface (z = 0 μm), the faradaic current density associated with charge transfer was described by the Butler-Volmer relation: [4]

$$i_{pos} = i_{0,pos}\left(e^{\frac{\alpha_{pos}F\eta}{RT}} - e^{-\frac{(1-\alpha_{pos})F\eta}{RT}}\right) \qquad \text{(S1)}$$

where i_{pos} is the faradaic current density at the LCO electrode, $i_{0,pos}$ is the corresponding exchange current density, α_{pos} is the charge-transfer coefficient, n is the number of transferred electrons, F is Faraday's constant, R is the gas constant, T is the absolute temperature, and η is the overpotential.

The concentration-dependent exchange current for the LCO electrode was evaluated using the following kinetic expression:

$$i_{0,pos} = a_F k_{pos}\left[\left(\frac{c_{Li,max} - c_{Li}}{c_{Li,max} - c_{Li,min}}\right)\frac{c_{Li^+}}{c_{0,Li^+}}\right]^{\alpha_{pos}}\left[\frac{(c_{Li} - c_{Li,min})}{(c_{Li,max} - c_{Li,min})}\right]^{1-\alpha_{pos}} \qquad \text{(S2)}$$

where a_F is the effective roughness factor defined in the main text, k_{pos} is the positive-electrode reaction-rate constant, $c_{Li,max}$ and $c_{Li,min}$ are the maximum and minimum Li^+ concentrations in LCO, [5] c_{0,Li^+} is the initial electrolyte concentration, and c_{Li} and c_{Li^+} are the local mobile Li^+ concentrations in LCO and the electrolyte, respectively.

These interfacial relations were coupled to the species-transport equations using the parameters in Tables S1 and S2; the reference-electrode expressions and all boundary conditions are summarized in Tables S3 and S4, respectively.

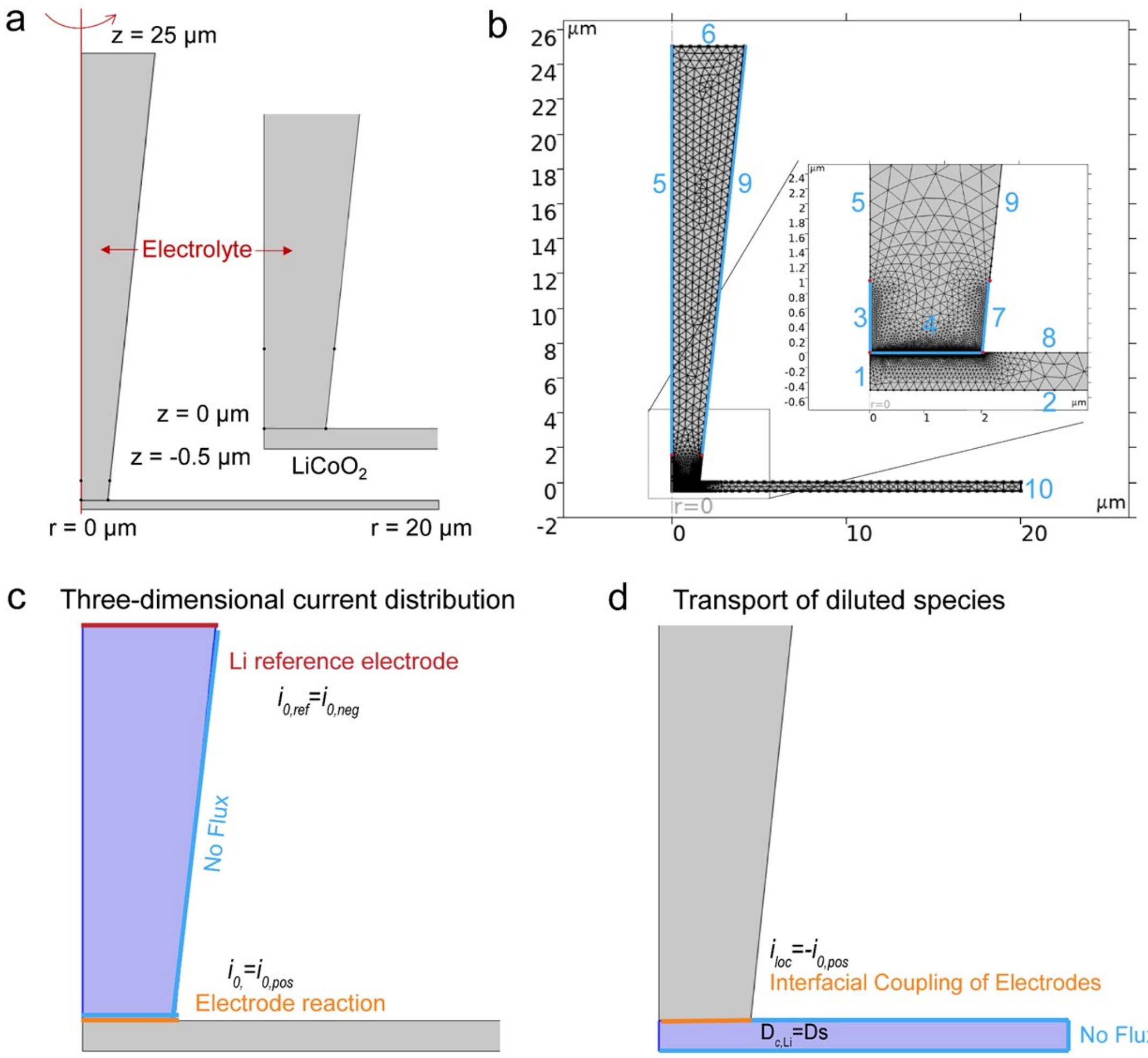

Figure S11. (a) 2D axisymmetric geometric model of the SECCM electrolyte droplet and the LCO substrate. (b) Finite element mesh layout, with labeling of all the boundaries for simulating the SECCM experiment. (c) Boundary conditions for the Tertiary Current Distribution interface applied to the electrolyte domain. (d) Domain and boundary conditions for the Transport of Diluted Species interface applied to the LCO solid domain, highlighting the interfacial coupling of the electrodes.

Evolution of concentration profiles during the electrochemical scanning of the LCO film on Day 0

By inputting the extracted a_F and *Ds* corresponding to the Day 0 conditions into the numerical model, we simulated the concentration profiles at various applied potentials during the anodic and cathodic scans, as plotted in Figure S12. These profiles explicitly visualize the Li ion profile from the conical electrolyte to the LCO electrode domain during the voltametric scanning.

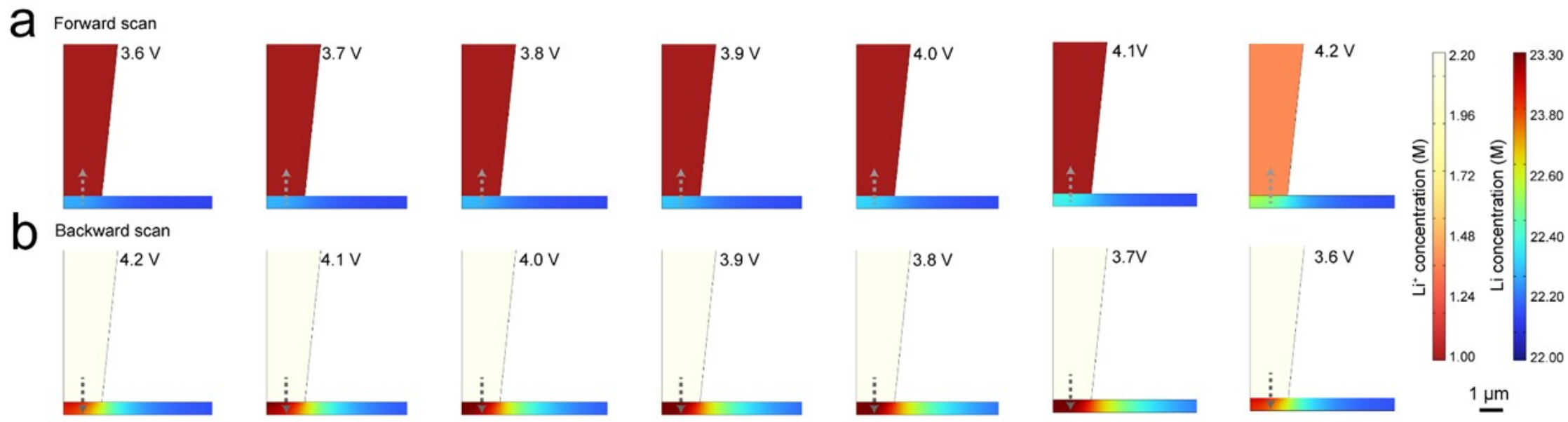

Figure S12. Simulated concentration profiles of the LCO film at various applied potentials during the (a) forward (anodic) scan and (b) backward (cathodic) scan under the Day 0 conditions.

Simulated CV curves of different microdomains under single variable control

To decouple the respective effects of a_F and Ds on the localized electrochemical responses, the CV curves for the R1, R2, and R3 microdomains were simulated using a control variable method. The simulations analyzed the independent variation of Ds under a constant a_F, and the independent variation of a_F under a constant Ds. This analytical approach enables the separation and extraction of characteristic electrochemical responses driven exclusively by the evolution of a single parameter.

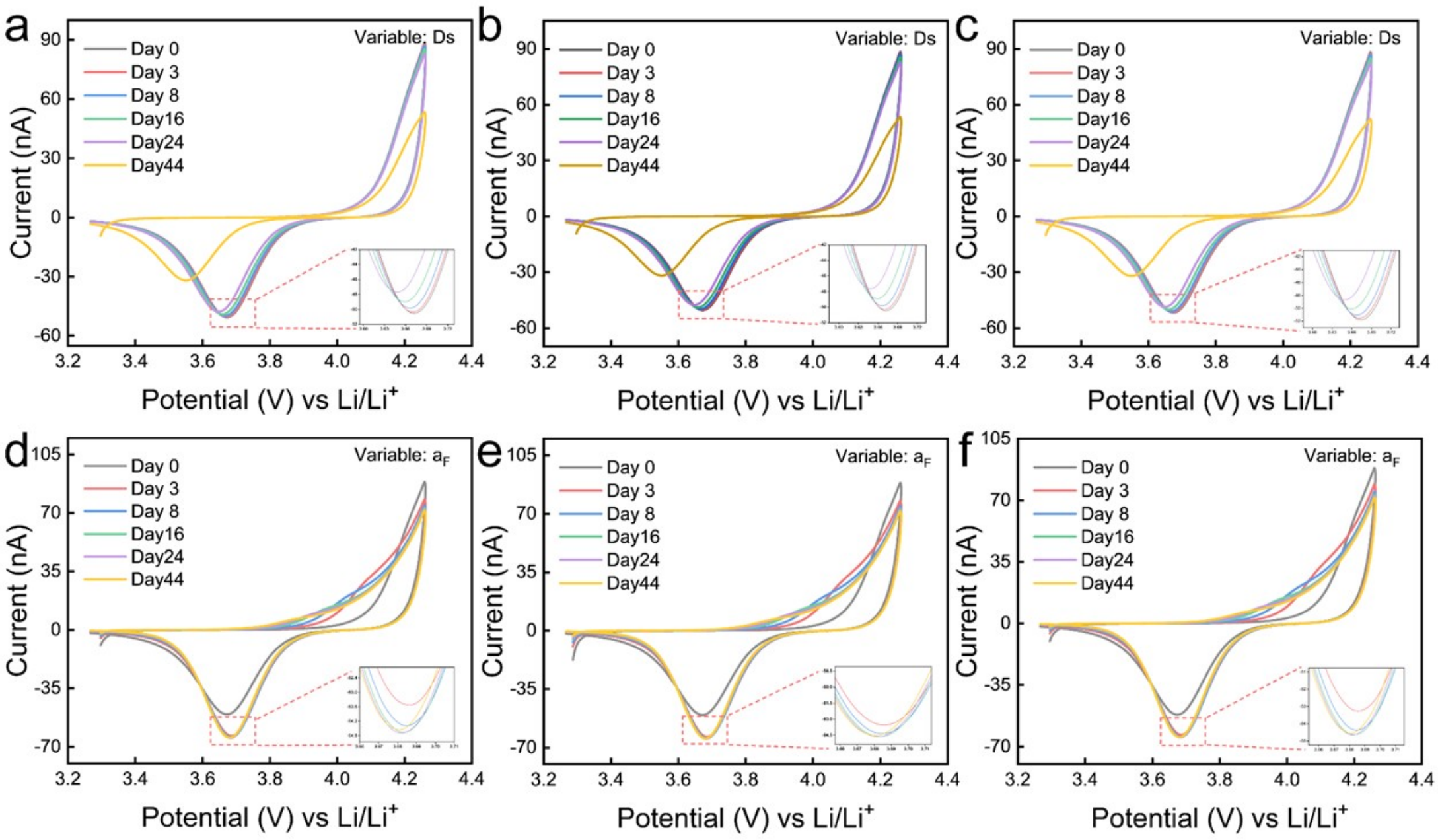

Figure S13. Simulated CV curves of the (a) R1, (b) R2, and (c) R3 microdomains with varying Ds under a constant a_F. Simulated CV curves of the (d) R1, (e) R2, and (f) R3 microdomains with varying a_F under a constant Ds. The insets present the magnified cathodic peak regions.

Simulated cathodic peak potentials and peak currents corresponding to single variable variations

The E_p^c and i_p^c values were extracted from the simulated CV curves in Figure S13. Figure S14 intuitively illustrates the independent impact of a single physical parameter variation on the electrochemical response characteristics.

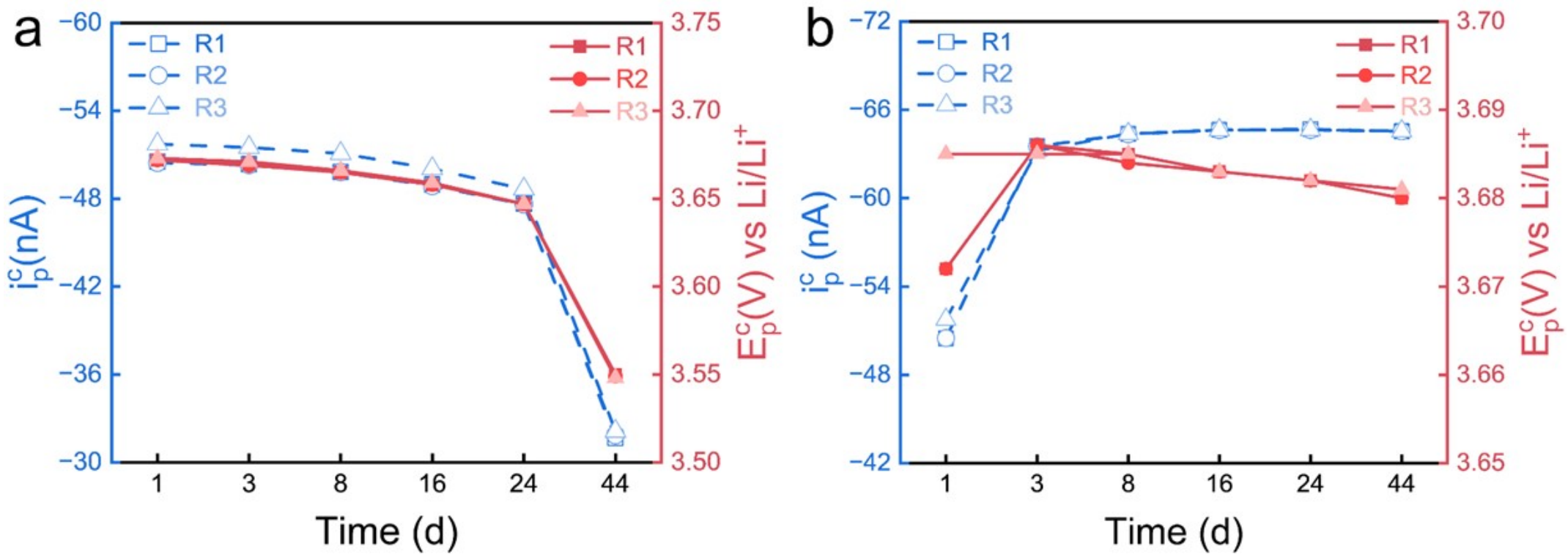

Figure S14. (a) Simulated cathodic peak potentials and peak currents of the R1, R2, and R3 microdomains with varying *Ds* under a constant a_F. (b) Simulated cathodic peak potentials and peak currents of the R1, R2, and R3 microdomains with varying a_F under a constant *Ds*.

Table S1. Physical parameters used in this work

To ensure the reliability of the multiphysics finite element simulations, the fundamental physical constants and kinetic parameters employed in the model are summarized in Table S1.

Parameter	
α_{neg}	0.5
α_{pos}	0.6
$c_{\mathrm{Li},max}$ [mM]	2.33×10^{4}
$c_{\mathrm{Li},min}$ [mM]	$c_{\mathrm{Li},max}$ / 2
$c_{0,\mathrm{Li},min}$ [M]	1
T [K]	298.15
k_{neg} [mol/m^2/s]	6×10^{-3}
k_{pos} [mol/m^2/s]	1×10^{-3}

Table S2. Parameter settings for roughness and surface composition

Considering the complex coupled effects of microcrack propagation, surface roughening, and chemical compositional changes during the actual aging process of the LCO material, two variables, *Ds* and a_F, are introduced into the model for equivalent phenomenological representation. To simplify the computational complexity, *Ds* is formulated as a time-dependent linear decay function. Meanwhile, a_F is defined as the sum of two components: an exponential term representing the kinetic growth of microcracks (a_{crack}) and a surface roughness factor (a_{RF}) derived directly from the experimental AFM measurements. The detailed mathematical relationships are summarized in Table S2.

Specifically, the AFM-derived roughness factor a_{RF}, which contributes to a_F, was obtained by dividing the reconstructed three-dimensional surface area by its projected area. For each 5 μm × 5 μm AFM image, three randomly selected 4 μm × 4 μm regions were reconstructed from the x, y, and z coordinates by Delaunay triangulation, and the mean ratio $a_F = A_{real}/A_{proj}$ (A_{proj}= 16 μm^2) was used. The phenomenological crack term a_{crack} was described by an exponential function to capture its nonlinear evolution. Similarly, D_s was treated as an empirical transport parameter whose decrease from Day 0 to Day 44 was described by a linear time-dependent function. This formulation reproduced the progressive cathodic peak-potential shift.

Parameter	
Ds_0 [m²/s]	9.85×10^{-13}
D_{fin} [m²/s]	3.15×10^{-14}
Ds	$Ds_0+(D_{fin}-Ds_0)\frac{t_{days}}{44}$
$D_{\mathrm{Li},ion}$ [m²/s]	1.2×10^{-6}
D_n	$D_{\mathrm{Li},ion}/2.5$
a_{RF}	1.027632
a_{crack}	$50\times(1-e^{-0.01t_{days}})$
a_F	$a_{RF}+a_{crack}$

Table S3. Definitions of electrochemical variables

The electrochemical variables governing the positive ($i_{0,pos}$) and negative ($i_{0,neg}$) interfacial kinetics are defined to facilitate the subsequent multiphysics coupling calculations. These mathematical formulations incorporate the local concentration gradients and a_F to quantitatively describe the dynamic charge transfer processes at the solid-liquid interfaces.

Variable	
$i_{0,pos}$	$k_{pos}(\frac{(c_{\mathrm{Li},max}-c_{\mathrm{Li}})c_{\mathrm{Li},ion}}{c_{0,\mathrm{Li},max}(c_{\mathrm{Li},max}-c_{\mathrm{Li},min})})^{\alpha_{pos}}(\frac{c_{\mathrm{Li}}-c_{\mathrm{Li},min}}{c_{\mathrm{Li},max}-c_{\mathrm{Li},min}})^{1-\alpha_{pos}}\mathrm{a}_F$
$i_{0,neg}$	$k_{neg}(\frac{c_{\mathrm{Li},ion}}{c_{0,\mathrm{Li},ion}})a_F$

Table S4. Boundary conditions used in the finite element model

Based on the geometrical boundaries delineated in Figure S11b, specific boundary conditions were applied to the finite element model. The Tertiary Current Distribution interface was assigned to the SECCM electrolyte domain to simulate Li^+ transport and potential gradients. Concurrently, the Transport of Diluted Species interface was applied to the solid LCO domain to model the dynamic evolution of the solid-state Li concentration.

Boundary	Boundary conditions
1, 3, 5	2D Axis of symmetry
2, 7, 8, 9, 10	No flux
4	Electrode surface: $i_{ioc,\exp r} = i_{0,pos}(e^{\frac{\alpha_{pos}F\eta}{RT}} - e^{\frac{-0.5F\eta}{RT}})$<br>Reaction coefficient： $R_i = \frac{v_i i_{0,pos}}{nF}$
6	Reference reaction: $i_{ioc,\exp r} = i_0(e^{\frac{\alpha_{pos}F\eta}{RT}} - e^{\frac{-0.5F\eta}{RT}})$<br>$i_0 = i_{0,neg} \prod_{i:v_1>0} (\frac{c_i}{c_{i,ref}})^{0.5v_i/n} \prod_{i:v_1>0} (\frac{c_i}{c_{i,ref}})^{-0.5v_i/n}$

References

1. C. M. Julien, A. Mauger and O. M. Hussain, *Materials*, 2019, **12**, 2687.
2. Z. Benzarti, J. D. Castro, E. Carneiro, L. Pacheco, N. Duarte, S. Carvalho, R. Serra, A. Cavaleiro, C. Alves and S. Cruz, *Materials*, 2025, **18**, 1217.
3. K. N. Wood and G. Teeter, *ACS Appl. Energy Mater.*, 2018, **1**, 4493-4504.
4. K. Iliev, A. R. Gizzatullin, A. A. Filimonova, N. D. Chichirova and I. H. Beloev, *Energies*, 2023, **16**, 7265.
5. D. Zhang, S.-X. Tang, L. D. Couto, Z. Wei and V. Viswanathan, *Automatica*, 2025, **173**, 112092.
